# Clonal Hematopoiesis of Indeterminate Potential and Cardiometabolic Disease: Challenges, Controversies and Future Perspectives

**DOI:** 10.3390/ijms27010233

**Published:** 2025-12-25

**Authors:** Ioanna A. Anastasiou, Dimitris Kounatidis, Natalia G. Vallianou, Eleni Rebelos, Irene Karampela, Maria Dalamaga

**Affiliations:** 1 Diabetes Center, First Department of Propaedeutic Internal Medicine, Medical School, National and Kapodistrian University of Athens, Laiko General Hospital, 11527 Athens, Greece; anastasiouiwanna@gmail.com (I.A.A.); dimitriskounatidis82@outlook.com (D.K.); elenirebelos@gmail.com (E.R.); 2 Department of Pharmacology, National and Kapodistrian University of Athens, 11527 Athens, Greece; 3 First Department of Internal Medicine, Sismanogleio General Hospital, 15126 Athens, Greece; natalia.vallianou@gmail.com; 4 Turku PET Centre, University of Turku, 20014 Turku, Finland; 5 Department of Clinical and Experimental Medicine, University of Pisa, 56126 Pisa, Italy; 6 Second Department of Critical Care, Attikon General University Hospital, Medical School, National and Kapodistrian University of Athens, 12462 Athens, Greece; eikaras1@gmail.com; 7 Department of Biological Chemistry, Medical School, National and Kapodistrian University of Athens, 11527 Athens, Greece

**Keywords:** aging, atherosclerosis, cardiometabolic, cardiovascular, clonal hematopoiesis, clonal hematopoiesis of indeterminate potential, CHIP, diabetes, inflammation, obesity

## Abstract

Clonal hematopoiesis of indeterminate potential (CHIP) is defined by the expansion of hematopoietic stem cells harboring leukemogenic mutations in the absence of overt malignancy. Strongly associated with advancing age, CHIP is detected by next-generation sequencing of peripheral blood in more than 20% of individuals over 80, most commonly through mutations in *DNMT3A*, *TET2*, *ASXL1*, and *PPM1D*. While CHIP confers over a four-fold increased risk of hematologic malignancy, it has recently emerged as a key determinant of cardiometabolic health. Epidemiological data indicated a 40% higher cardiovascular disease (CVD) risk events and a 34% increase in all-cause mortality among CHIP carriers, with specific mutations and larger clone sizes conferring greater cardiovascular burden. Preclinical studies have shown that macrophages deficient in *TET2* or *DNMT3A* drive interleukin (IL)-1β/IL-6 inflammasome activation, thereby promoting atherosclerosis and metabolic dysfunction, whereas the *JAK2V617F* mutation accelerates thrombosis. CHIP integrates into a broader network of dysregulation encompassing adiposity and inflammaging, which underlies its association with diverse comorbidities, including type 2 diabetes (T2D), chronic kidney disease (CKD), and chronic obstructive pulmonary disease (COPD). Multi-omics approaches have identified epigenetic and proteomic signatures correlated with CHIP expansion, providing potential biomarkers for risk stratification. Despite growing evidence of its systemic impact, CHIP screening remains limited to research settings. Emerging therapeutic strategies, including inflammasome inhibition, STING modulation, and epigenetic restoration, highlight its potential as a modifiable risk factor. This narrative review synthesizes current epidemiological, mechanistic, and translational insights, framing CHIP as an emerging causal factor in cardiometabolic disease and as a promising target for precision medicine in aging populations.

## 1. Introduction

Hematopoiesis is the process by which blood cells such as red blood cells (RBC), white blood cells (WBC), and platelets are produced from hematopoietic stem cells (HSCs) in the bone marrow [1]. HSCs can self-renew or differentiate into multipotent progenitors (MPPs), which further specialize into myeloid and lymphoid lineages [2]. Myeloid progenitors (CMPs) give rise to cells including granulocytes, monocytes, megakaryocytes, and erythrocytes, while lymphoid progenitors produce T and B lymphocytes, natural killer (NK) cells, and dendritic cells (DCs) [3]. Throughout this differentiation, somatic mutations inevitably occur, including base substitutions, small insertions or deletions, and larger structural changes, all of which tend to accumulate with age. With advancing age, numerous somatic mutations accumulate in HSCs, some of which can confer a growth advantage leading to clonal expansion, a phenomenon known as clonal hematopoiesis (CH) [4,5].

Clonal hematopoiesis of indeterminate potential (CHIP) is defined by the expansion of a hematopoietic stem cell carrying a leukemogenic mutation in individuals without evidence of hematologic malignancy, dysplasia, or cytopenia [6,7]. CHIP is detected in about 5–10% of adults aged 60–69 years and in >20% of those aged ≥80 years [8,9,10]. Many CHIP carriers remain asymptomatic, similar to those with monoclonal gammopathy of undetermined significance (MGUS) [11]. However, individuals with CHIP face up to a 13-fold higher risk of developing hematologic malignancies, with acute myeloid leukemia (AML) being the most prominent. Furthermore, CHIP is correlated with a 1.4-fold increase in all-cause mortality, despite the relatively modest annual progression rate to overt blood cancer of only 0.5–1% [12,13,14,15]. Evidence also links CHIP to a higher risk of therapy-related myeloid neoplasms (MN), particularly following chemotherapy or radiation exposure, with tumor protein p53 (*TP53*) mutations playing a central role in predisposing to therapy-related leukemia and myelodysplastic syndromes (MDS). In hematopoietic stem cell transplant recipients, CHIP is further linked to adverse clinical outcomes and increased mortality [16,17,18].

**Figure 1:**
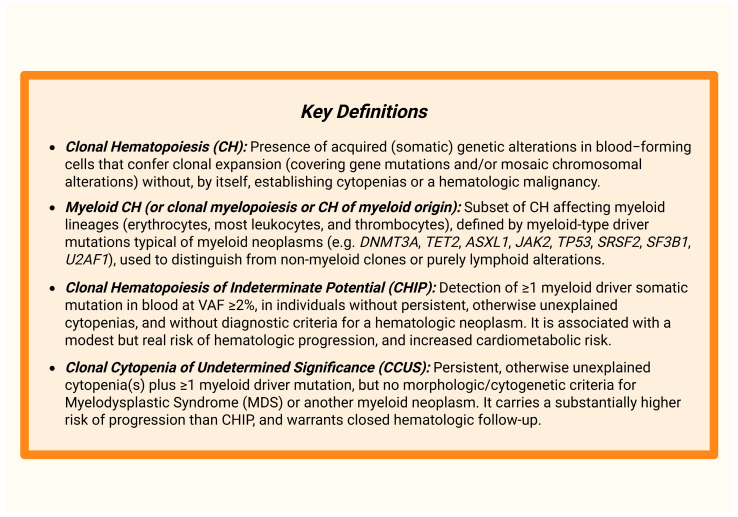
Key definitions of clonal hematopoiesis and related entities. The traditional diagnostic cut-off for CHIP is a VAF ≥ 2%, although emerging evidence indicates that smaller clones may also confer clinical relevance. Abbreviations: *ASXL1*: additional sex combs like 1; *DNMT3A*: DNA (cytosine-5)-methyltransferase 3 alpha; *JAK2*: janus kinase 2; *SF3B1*: splicing factor 3b subunit 1; *SRSF2*: serine/arginine-rich splicing factor 2; *TET2*: tet methylcytosine dioxygenase 2; *TP53*: tumor protein p53; *U2AF1*: U2 small nuclear RNA auxiliary factor 1; VAF: variant allele frequency. Created in BioRender. Kounatidis, D. (2025) https://BioRender.com/tou4j28 (accessed on 30 September 2025).

Approximately 80% of CHIP cases are driven by recurrent somatic mutations across distinct functional gene categories. The most frequently mutated genes are those involved in epigenetic regulation, including *DNA* (*cytosine-5*)*-methyltransferase 3 alpha* (*DNMT3A*), *ten-eleven translocation 2* (*TET2*), and *additional sex combs-like 1* (*ASXL1*). Mutations are also common in DNA damage response genes, such as *protein phosphatase*, *Mg*^2+^/*Mn*^2+^*-dependent 1D* (*PPM1D*) and *TP53*. Other affected categories include signaling regulators, exemplified by *Janus kinase 2* (*JAK2*), and mRNA spliceosome components, such as *splicing factor 3B subunit 1* (*SF3B1*) and *serine/arginine-rich splicing factor 2* (*SRSF2*) [19]. Traditionally, CHIP has been defined by the presence of a driver mutation with a variant allele frequency (VAF) of at least 2%, a threshold typically confirmed using deep sequencing. Nevertheless, advances in high-throughput sequencing technologies now enable the reliable detection of clones at much lower VAFs. The clinical significance of these very small clones remains uncertain, as their contribution to disease risk is influenced by biological heterogeneity, mutation context, and clone-specific effect size, e.g., whether mutations disrupt DNA methylation programs (*DNMT3A*, *TET2*), alter chromatin regulation (*ASXL1*), impair DNA damage responses (*TP53*, *PPM1D*) or affect mRNA splicing (*SF3B1*, *SRSF2*) [20]. Figure 1 presents key definitions, providing a visual reference for the terminology discussed throughout the text.

In addition to its well-established role in hematologic malignancies, a growing body of evidence implicates CHIP in diverse CVD phenotypes, including coronary artery disease (CAD), heart failure (HF), and ischemic stroke [13]. This link is influenced both by the age-associated expansion of mutant HSC clones and by their capacity to drive systemic pro-inflammatory signaling. Mechanistically, mutant clones drive increased production of pro-inflammatory cytokines such as interleukin (IL)-1β, IL-6, and tumor necrosis factor-α (TNF-α), with activation of the NLR family pyrin domain containing 3 (NLRP3) inflammasome serving as a central pathway [11]. In an exploratory analysis of the Canakinumab Anti-inflammatory Thrombosis Outcomes Study (CANTOS), IL-1β inhibition with canakinumab reduced recurrent cardiovascular events, even in individuals without CHIP, supporting the hypothesis that IL-1β signaling is a key mediator of atherothrombosis and likely contributes to CHIP-associated CVD risk [21,22]. Lifestyle interventions, such as adherence to a healthy diet, may further mitigate CVD risk in CHIP carriers by diminishing systemic inflammation [23].

Experimental and epidemiological studies indicate that the cardiovascular impact of CHIP is mutation-specific, with *TET2* and *JAK2* mutations showing the most potent associations with adverse outcomes [24]. Consistent with its role in hematologic malignancies, VAFs above 2% are generally regarded as clinically meaningful, since larger clones are associated with stronger inflammatory responses, and higher risk of adverse cardiac events. Nevertheless, emerging data suggest that even smaller clones may confer measurable risk [25,26]. Longitudinal analyses further demonstrated that high-VAF clones frequently expand over time, sometimes by more than 50%, a dynamic that correlates with increased incidence of CVD events and progressive disease burden. Lastly, CHIP has been associated with accelerated epigenetic aging, with affected individuals exhibiting biological age markers that exceed their chronological age. This observation suggests that CHIP contributes not only to specific disease phenotypes but also to a broader age-related decline, with implications for morbidity and mortality across multiple organ systems [27].

Beyond cardiovascular outcomes, CHIP has been associated with metabolic disorders, including type 2 diabetes (T2D) and metabolic dysfunction-associated steatohepatitis (MASH), conditions in which chronic low-grade inflammation, insulin resistance (IR), and obesity exhibit key roles. Mechanistically, CHIP-related mutations skew HSC differentiation toward myeloid lineages and promote macrophage activation, thereby amplifying adipose tissue and hepatic inflammation and contributing to metabolic dysfunction [28,29]. Chronic low-grade inflammation, IR, and obesity are central to this process, and obesity itself exacerbates CHIP expansion and its inflammatory consequences [30]. CHIP has also been linked to chronic inflammatory diseases, such as chronic obstructive pulmonary disease (COPD), as well as infections with significant CVD burden, like coronavirus disease 2019 (COVID-19) and human immunodeficiency virus (HIV), where altered innate and adaptive immune responses may further reinforce systemic cytokine dysregulation [31].

While numerous reviews have addressed the role of CHIP in CVD, relatively few have examined its broader systemic impact across metabolic, inflammatory, and age-related conditions. Of note, the reciprocal relationship between CHIP and obesity, where inflammation promotes clonal expansion and, in turn, CHIP exacerbates metabolic dysfunction, remains underrecognized. This review seeks to elucidate the mechanistic links between CHIP and cardiometabolic disease, with an emphasis on inflammatory pathways. Furthermore, it aims to pinpoint therapeutic opportunities for mitigating CHIP-associated cardiovascular and cardiometabolic risks. Recognizing CHIP as a causal, systemic driver of cardiometabolic disease may transform risk prediction, biomarker discovery, and precision therapies in aging medicine. Given growing evidence of a bidirectional interplay between adiposity, inflammation, and clonal expansion, the relationship between CHIP and obesity represents a particularly novel and underexplored dimension of cardiometabolic risk.

## 2. Literature Search

For this narrative review, a comprehensive literature search was performed using the PubMed database, which was selected because it provides broad coverage of hematologic, cardiologic, and metabolic subjects as well as indexed clinical trials. The search strategy included the keywords “clonal hematopoiesis of indeterminate potential (CHIP)” and “cardiometabolic disorders,” limited to publications from the past 25 years, yielding 689 articles published between 2000 and December 2025. We restricted our search to peer-reviewed articles published in English.

We primarily included human observational (cohort, case–control, registry and Biobank-based) and interventional studies, as well as key meta-analyses and systematic reviews. Experimental work in animal models and in vitro systems was included when it provided mechanistic insight into CHIP-associated inflammation, atherosclerosis, metabolic dysfunction or organ-specific injury. Conference abstracts were considered only when they reported CHIP–cardiometabolic associations not yet available in full-text form. We excluded non-peer-reviewed material and articles not directly addressing CHIP or mutation-driven clonal hematopoiesis in the context of cardiovascular, metabolic, or inflammatory outcomes. The reference lists of selected studies were carefully examined to identify additional relevant publications, thereby complementing the PubMed search and improving completeness. Given the extensive volume of retrieved literature, not all pertinent studies could be discussed in detail within the scope of this review, while emphasis was placed on representative, recent and methodologically robust work.

Because this review was narrative rather than systematic, study selection was based on relevance and scientific contribution rather than exhaustive retrieval. This approach may introduce selection bias, although it enables integration of mechanistic, translational and epidemiologic evidence that may not conform to standardized systematic review frameworks.

## 3. CHIP: From Blood Cancer Risk to Cardiometabolic Disease

Although CHIP was initially identified as a precursor state for MN, accumulating evidence shows that its clinical impact extends well beyond hematologic malignancy. While progression risk varies by mutation type, clone size and mutational burden, the broader significance of CHIP lies in its systemic inflammatory footprint, which contributes to cardiometabolic disease, metabolic dysfunction and age-related multimorbidity [14,20].

The risk of malignant progression is heterogeneous. The clonal hematopoiesis risk score (CHRS) stratifies individuals into 10-year MN risk groups ranging from ~0.7% to >50%, depending on mutation category, mutation burden, and VAF, with validation across large biobanks [32]. Isolated *DNMT3A* mutations generally confer lower malignant potential, whereas prospective CCUS studies have shown that mutation number is the strongest predictor of progression [33].

Therapy-related MN (t-MN) frequently arise from pre-existing CH clones, particularly TP53, detectable years before transformation, reframing t-MN as clonal selection rather than direct mutagenesis [34]. In hematopoietic stem cell transplantation, both residual recipient CH and donor-derived CH affect outcomes after autologous and allogeneic HSCT, supporting routine CH evaluation [35]. Clinically, hematology referral is recommended when CHIP coexists with cytopenias, rising VAF, ≥2 mutations, high-risk genotypes, or prior cytotoxic exposure, with follow-up using serial CBCs and periodic NGS. These recommendations are consistent with emerging expert consensus statements and multi-center position papers that advocate risk-adapted monitoring in individuals with CCUS, high-VAF clones, or *TP53*/spliceosomal mutations, even though formal international guidelines are not yet established [33].

CHIP is also seen in non-malignant hematologic disorders such as acquired aplastic anemia, where ~50% of patients harbor CH-related mutations. Among these, *BCOR/BCORL1* variants tend to remain stable and predict favorable responses to immunosuppressive therapy, whereas mutations in *DNMT3A* and *ASXL1* are associated with clonal expansion, progression to MDS/AML, and reduced survival [36].

These immune-related patterns are clinically relevant because aplastic anemia illustrates how CHIP-associated mutations reshape hematopoiesis and immune surveillance, themes that recur in the systemic effects of CHIP across cardiometabolic disorders through chronic inflammation and altered immune function [37].

In recent years, the relevance of CHIP to human health has extended beyond malignancy to include CVD and associated metabolic comorbidities. Findings suggest that metabolic dysfunction may modulate clonal behavior, as hyperglycemia, oxidative stress and chronic low-grade inflammation may increase DNA damage and hematopoietic stress, thereby promoting selective expansion of mutant HSC clones. This mechanism provides a biological rationale for the observed epidemiologic overlap between CHIP, diabetes and cardiometabolic disease [38].

Further important insights into the relationship between CHIP and various aspects of human health come from a recent meta-analysis of 88 studies involving nearly 500,000 individuals [39]. This analysis showed that CHIP carriers had a markedly increased risk of hematologic cancers (HR 4.28; 95% CI, 2.29–7.98). Notably, the investigation highlighted CHIP as a risk factor for CVD, revealing associations with composite cardiovascular events (HR 1.40; 95% CI, 1.19–1.65), CAD (HR 1.76; 95% CI, 1.27–2.44), stroke (HR 1.16; 95% CI, 1.05–1.28), and HF (HR 1.27; 95% CI, 1.15–1.41). Although CHIP was linked to increased all-cause mortality (HR 1.34; 95% CI, 1.19–1.50), no significant association was observed with cardiovascular mortality (HR 1.09; 95% CI, 0.97–1.22), while larger clone size was associated with higher risk (HR 1.31; 95% CI, 1.12–1.54). Importantly, isolated *DNMT3A* mutations did not significantly affect the risk of myeloid malignancies or all-cause mortality. Heterogeneity across studies appeared to stem from differences in CHIP definitions, detection methods, and population characteristics. Overall, these findings indicate that CHIP is linked to a spectrum of clinical outcomes, with clone size, gene-specific mutations, and patient-level factors shaping disease risk [39]. Within this context, the relationship between CHIP and cardiometabolic disease emerges as particularly relevant and will be explored in the following sections. A concise overview of key in vivo, in vitro and human studies relevant to CHIP and its association with cardiometabolic disease, together with commonly used diagnostic approaches, is summarized in Table 1.

## 4. CHIP and Cardiovascular Diseases

A growing body of evidence has underscored the strong association between CHIP and CVDs. This relationship appears to be bidirectional, as traditional cardiovascular risk factors increase the mutational burden in CHIP, while CHIP itself contributes to the incidence and prognosis of various forms of CVD [51,52]. The presence of CHIP in peripheral blood cells has been linked to a two-fold increased risk of Atherosclerotic CVD (ASCVD) in humans, as well as to accelerated atherosclerosis in murine models [53]. Recent studies have also highlighted the potential use of scoring systems to estimate all-cause and cardiovascular mortality in older adults with CH [49]. Both the type of mutation and the size of the clone appear to be critical determinants of cardiovascular risk, with inflammation and thrombosis identified as the main mechanisms driving atherosclerosis and CVD [52].

The following sections will review current evidence on the association between CHIP and major cardiovascular outcomes and will discuss the biological mechanisms that may underlie this relationship.

### 4.1. The Association of CHIP with Specific Cardiovascular Phenotypes

#### 4.1.1. CHIP and Coronary Artery Disease

In recent years, CHIP has emerged as a novel, non-traditional risk factor that influences not only the development and progression of CAD but also long-term clinical outcomes. In a recent study of 1142 patients undergoing coronary angiography, 18.4% were found to carry a CHIP mutation, which was associated with an increased risk of obstructive left main and left anterior descending coronary artery stenosis, particularly in individuals harboring *TET2* mutations [54]. Complementary data further showed that CHIP carriers had a 39% higher risk of mortality over a three-year follow-up (*p* < 0.001). Mutations in several genes, including *TET2*, *ASXL1*, *DNMT3A*, *JAK2*, *PPM1D*, *SF3B1*, *SRSF2*, and *U2AF1*, were each independently associated with an increased likelihood of death. Among these, *TET2* mutations were particularly notable, as they promoted a pro-atherogenic macrophage phenotype through upregulation of the low-density lipoprotein receptor (LDLR) and enhancement of inflammatory signaling. Therefore, epigenetic dysregulation is a key driver of adverse cardiovascular outcomes in CAD patients [55].

Collectively, these findings support the concept that CHIP-associated inflammation promotes atherosclerotic lesion complexity through macrophage-driven LDLR upregulation, enhanced cytokine signaling and altered lipid handling. Gene-specific effects, particularly involving *TET2* and *DNMT3A*, reinforce the notion that epigenetic dysregulation shapes vascular inflammation and plaque vulnerability.

Evidence also suggests that CHIP may affect long-term outcomes in patients undergoing coronary artery bypass grafting (CABG). A recent study demonstrated that CHIP mutations are relatively common in this population and confer a higher risk of mortality. Over a median follow-up of six years, CHIP defined by a VAF ≥ 2% was not significantly associated with major adverse cardiac and cerebrovascular events, non-fatal ischemic stroke, or myocardial infarction (MI). However, it was independently associated with increased risks of both all-cause mortality (aHR 1.73, 95% CI 1.08–2.78) and cardiovascular mortality (aHR 2.58, 95% CI 1.47–4.55), after multivariable adjustment for age, sex, BMI, comorbidities (including diabetes, hypertension and dyslipidemia) and surgical risk factors. In contrast, smaller CHIP clones (VAF 0.1–2%) were not significantly associated with adverse long-term outcomes [56].

#### 4.1.2. CHIP and Heart Failure

Recent large-scale prospective studies have identified CHIP as an emerging risk factor for heart failure (HF). Specifically, sequence variations in *ASXL1*, *TET2*, and *JAK2* have been linked to a 25% increase in the risk of incident HF, with the impact of CHIP on HF incidence following an age-dependent pattern [57,58]. Moreover, patients carrying *DNMT3A* or *TET2* mutations experienced substantially worse long-term outcomes, including higher rates of mortality and HF-related hospitalization, with greater mutational burden correlating with progressively adverse prognosis [59]. Beyond incidence, CHIP has been implicated in cardiovascular mortality across both ischemic and non-ischemic HF with reduced ejection fraction (HFrEF). In a pivotal study by Pascual-Figal et al., *DNMT3A* or *TET2* mutations were independently associated with accelerated HF progression. Even after adjustment for conventional risk factors, carriers demonstrated significantly increased hazards for death (aHR 2.79; 95% CI, 1.31–5.92; *p* = 0.008), death or HF hospitalization (aHR 3.84; 95% CI, 1.84–8.04; *p* < 0.001), and HF-related death or hospitalization (aHR 4.41; 95% CI, 2.15–9.03; *p* < 0.001) [60].

CHIP may also play a role in HF with preserved ejection fraction (HFpEF). Specifically, *TET2*-related CHIP has been independently associated with a 2.4-fold increased risk of HFpEF, particularly in individuals exhibiting systemic inflammation, as indicated by C-reactive protein (CRP) levels ≥ 2 mg/L. Of note, no significant association was observed with incident HFrEF in this study [61]. Additionally, in individuals with dilated cardiomyopathy (DCM), the presence of CHIP has been correlated with higher risks of cardiac death (HR: 2.33; VAF cutoff 0.36%; 95% CI: 1.24–4.40) and all-cause mortality (HR: 1.72; VAF cutoff 0.06%; 95% CI: 1.10–2.69), independently of age, sex, left ventricular ejection fraction, and New York Heart Association (NYHA) functional class [62]. Mechanistically, mutations in epigenetic regulators such as *DNMT3A* and *TET2* enhance maladaptive cardiac remodeling by promoting macrophage-derived cytokine secretion, augmenting endothelial–fibroblast crosstalk and amplifying pro-fibrotic signaling pathways. These alterations align with experimental models in which mutant myeloid cells exacerbate myocardial inflammation, fibrosis and impaired ventricular recovery [63].

#### 4.1.3. CHIP and Stroke

Emerging evidence suggests that CHIP is associated with an increased risk of stroke, independent of traditional vascular determinants such as age and sex. Some data indicate that this association may be stronger for hemorrhagic than for ischemic stroke. Among ischemic stroke subtypes, *TET2* mutations appear to play a particularly important role; however, findings remain inconsistent regarding which subtype is most strongly linked to CHIP. For example, one study reported a stronger association with small-vessel ischemic stroke [64] whereas other data point towards a connection with cardioembolic stroke, supported by evidence linking CHIP to larger infarcts. In contrast, intracranial arterial stenosis has shown only marginal associations [65]. Beyond incidence, CHIP also carries prognostic significance. It has been independently associated with greater initial stroke severity, a higher risk of hemorrhagic transformation, and poorer 90-day functional outcomes [66]. Moreover, CHIP has been linked to long-term stroke recurrence, particularly in patients with a high atherosclerotic burden [67]. Incorporating CHIP into predictive models alongside established risk factors may improve ischemic stroke risk stratification, with larger clones conferring disproportionately higher hazards [68]. Notably, findings from Sun et al. demonstrated that in patients with diabetes, CHIP was significantly associated with CAD and HF, but not with stroke [69].

Taken together, although epidemiologic data consistently link CHIP to a spectrum of vascular outcomes, the precise mechanistic pathways remain incompletely understood, with ongoing uncertainty regarding mutation-specific effects, vascular bed heterogeneity and the extent to which inflammation versus thrombosis predominates in different phenotypes.

#### 4.1.4. CHIP and Peripheral Artery Disease

Data on the association between CHIP and peripheral artery disease (PAD) remain extremely limited. A single pilot study involving 31 individuals with PAD undergoing open surgical procedures revealed that somatic CH mutations were identified in 45% of participants, with *TET2* (55%) and *DNMT3A* (40%) representing the most frequently affected genes. Notably, approximately 90% of mutations detectable in peripheral blood were also present within atherosclerotic plaques, and some of these variants were additionally identified in perivascular adipose tissue and subcutaneous tissue [70].

#### 4.1.5. CHIP and Other Cardiovascular-Related Outcomes

Evidence suggests that CHIP may constitute a novel risk factor for new-onset cardiac arrhythmias. Using data from 410,702 individuals in the UK Biobank, Schuermans et al. demonstrated that both any CHIP and large CHIP clones were associated with modest, yet statistically significant, increases in the risk of supraventricular arrhythmias, bradyarrhythmias, and ventricular arrhythmias. These associations were independent of underlying CAD and HF and were heterogeneous across arrhythmia subtypes, with the strongest effect observed for cardiac arrest. Gene-specific analyses indicated elevated arrhythmia risk for most CHIP driver genes except *DNMT3A*, while large CHIP was also linked to a 1.31-fold higher likelihood of being in the top quintile of myocardial fibrosis assessed by cardiac magnetic resonance imaging (*p* = 0.009) [26]. Mendelian randomization data further supported that genetic predisposition to CHIP increases the risk of atrial fibrillation (AF) [25]. Interestingly, CHIP may also predispose to inflammatory cardiac conditions, particularly myocarditis and pericarditis, in middle-aged populations. In a population-based cohort of 335,426 individuals, both overall CHIP and larger clone sizes were linked to elevated risks of these outcomes, with multivariable-adjusted hazard ratios of 1.75 (*p* = 0.01) and 2.07 (*p* = 0.003), respectively. The associations were most pronounced for *DNMT3A*- and *TET2*-related CHIP, and the strength of the relationship with myocarditis and pericarditis exceeded that observed for other cardiovascular conditions such as CAD or HF. Furthermore, CHIP carriers exhibited a 1.27-fold increased risk of developing non-cardiac immune-mediated inflammatory diseases (*p* < 0.001) [71]. Table 2 presents clinical evidence linking CHIP to diverse CVD phenotypes.

### 4.2. CHIP–Atherosclerosis Crosstalk and Inflammaging

Sequencing studies have established a robust clinical link between mutation driven CH and atherosclerosis. Inflammaging refers to the chronic, low-grade, sterile inflammatory state that develops with aging and contributes to multiple age-related diseases, including atherosclerosis, HF, metabolic dysfunction and immune dysregulation [72]. Earlier investigations indicated that somatic mutations in HSCs can influence vascular properties, while more recent models suggest that atherosclerosis itself may promote clonal expansion. These findings point to a bidirectional relationship, although the mechanistic basis remains incompletely understood [73].

#### 4.2.1. The Impact of CHIP on Atherosclerosis Development

The contribution of CHIP to atherosclerosis varies by driver mutation and reflects distinct molecular programs. *TET2* loss enhances NF-κB-dependent IL-1β transcription in macrophages, amplifying NLRP3 inflammasome activity and promoting vascular inflammation, whereas *JAK2V617F* activates STAT-mediated cytokine signaling, increases neutrophil extracellular trap (NET) formation, and augments thrombo-inflammatory responses. In contrast, DNMT3A-mutant clones generally exhibit lower pro-inflammatory potential, although context-dependent effects have been observed. Inflammation becomes more pronounced as clonal size increases, since higher VAF are linked to elevated circulating pro-inflammatory cytokines. Larger clones therefore seem to associate with stronger systemic inflammatory responses and with a greater risk of cardiovascular-related outcomes, like myocardial infarction (MI), stroke, and cardiovascular death [13,14].

NLRP3 has emerged as a key inflammatory regulator in this process. In experimental models, loss of *TET2* in hematopoietic cells accelerates atherosclerosis in LDLR deficient mice through NLRP3 mediated IL-1β and IL-6 signaling. The phenotype shows a gene dosage dependent effect and can be attenuated by NLRP3 inhibition [40,53]. These findings have direct therapeutic implications. Targeting IL-1β with agents such as canakinumab attenuates NLRP3-driven inflammation, while small-molecule NLRP3 inhibitors (e.g., DFV890) show the ability to blunt mutant myeloid cell activation in early-phase studies. Collectively, these data support NLRP3 and downstream IL-1 family signaling as actionable pathways in CHIP-associated atherosclerosis [74,75].

Beyond its role in atherosclerosis, macrophage-mediated NLRP3 activation has been implicated in the worsening of HF, promoting myocardial fibrosis and impairing cardiac function, even in the absence of overt CAD [76]. Additionally, recent research supports that *DNMT3A* mutant monocytes may display upregulation of inflammatory and phagocytic pathways, while CD4 positive T cells and natural killer (NK) cells carrying mutations show increased activation and effector signatures. In this context, enhanced paracrine communication between mutant and wild type cells provides an additional mechanism by which CH can accelerate the progression of HF [77]. Murine models expressing *JAK2V617F* demonstrate a distinct prothrombotic phenotype. NET formation is enhanced, leading to venous thrombosis, while myeloid restricted expression of *JAK2V617F* promotes arterial thrombosis through platelet activation and hematocrit elevation. The resulting NET mediated intimal injury contributes to superficial erosion, a mechanism responsible for approximately 25% of ACS [44,78,79]. The vascular consequences of CH may extend to the coronary microcirculation. Patients with coronary microvascular dysfunction, which reflects impaired function of the small intramyocardial vessels, are more likely to carry CHIP associated mutations. In this setting, nearly one third of major adverse cardiovascular events (MACE) appear to be mediated by CH [80].

Beyond the heart, CHIP may accelerate cerebrovascular aging by promoting inflammation and oxidative stress that impair endothelial integrity, reduce cerebral blood flow, and disrupt the blood–brain barrier. These changes are implicated in cerebral small vessel disease and may contribute to vascular cognitive impairment and age-related cognitive decline [81]. CH has also been associated with PAD, with studies in LDLR deficient chimeric mice showing that TP53 mutant hematopoietic cells promote plaque growth and macrophage expansion [82]. Interestingly, carriers of CHIP may exhibit accelerated epigenetic aging, often presenting with a biological age exceeding chronological age. This phenomenon may contribute to vascular dysfunction and help explain the elevated incidence of atherosclerosis and cardiovascular events observed in these individuals [27].

#### 4.2.2. Inflammation-Driven Clonal Expansion

The chronic inflammatory environment present in atherosclerosis, marked by elevated cytokines such as IL-1β, IL-6, TNF-α, and interferon-gamma (IFN-γ), promotes oxidative stress and DNA damage in HSCs, particularly given their high turnover rates required to sustain lifelong hematopoiesis [83]. These cytokines not only drive genomic instability but also impair DNA repair mechanisms, thereby facilitating the accumulation of somatic mutations in HSCs and progenitor cells. Dysregulation of the DNA damage response, together with persistent inflammatory signaling, disrupts HSC maintenance and self-renewal, enabling the emergence and clonal expansion of mutated hematopoietic populations. Experimental evidence shows that inflammation-driven stress favors the expansion of *DNMT3A-* and *TET2*-mutant clones, especially under conditions of chronic infection or exposure to cytokines such as IFN-γ [46,84]. These mutant clones, in turn, produce increased levels of pro-inflammatory cytokines, amplifying systemic inflammation and contributing to atherosclerotic plaque progression and instability, thereby establishing a self-perpetuating vicious cycle [46].

Advanced atherosclerosis not only arises from increased inflammation but also actively promotes HSC proliferation and mutation accumulation through higher hematopoietic demand. In this context, elevated bone marrow activity facilitates the emergence of CHIP-associated clones, particularly those carrying *TET2* and *DNMT3A* mutations, which further intensify inflammatory responses and accelerate disease progression [37]. Importantly, systemic inflammation, as reflected by elevated levels of C-reactive protein (CRP), shows a strong correlation with CHIP, underscoring the central role of inflammation in both the development and clinical consequences of CH [85].

In summary, atherosclerosis and CHIP form a self-perpetuating cycle in which inflammation drives HSC proliferation and mutation accumulation, while mutant clones, particularly those with *TET2* and *DNMT3A* alterations, boost pro-inflammatory signaling and accelerate vascular dysregulation. High HSC turnover and impaired DNA repair increase susceptibility to genomic instability, and external factors like chronic infections can further promote clonal expansion via cytokine pathways [46,86]. This interplay underscores the central role of the inflammatory microenvironment and highlights the potential of targeted strategies to modulate inflammation, preserve HSC integrity, and limit clonal evolution, ultimately reducing CHIP-associated cardiovascular risk.

**Figure 2:**
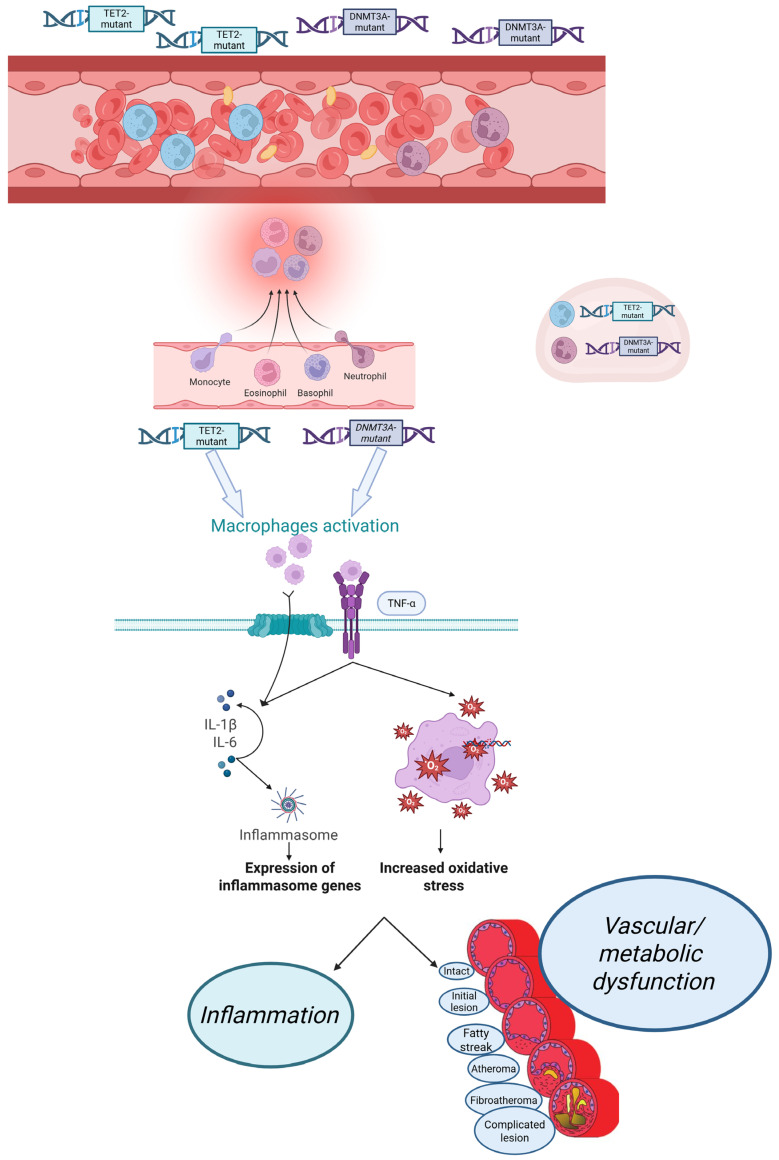
The core inflammaging pathways linking CHIP, cytokine activation and vascular/metabolic dysfunction. Inflammatory signals stimulate these clones to grow further, creating a cycle of increased inflammation. This process drives CHIP progression and increases the risk of related diseases. Abbreviations: *DNMT3A*: DNA (cytosine-5)-methyltransferase 3A; IL-1β: interleukin-1β; IL-6: interleukin-6; *TET2*: ten-eleven translocation 2; TNF-α: tumor necrosis factor-alpha; Created in BioRender. Anastasiou, I. (2025) https://BioRender.com/c00mfbd (accessed on 3 October 2025).

**Figure 3:**
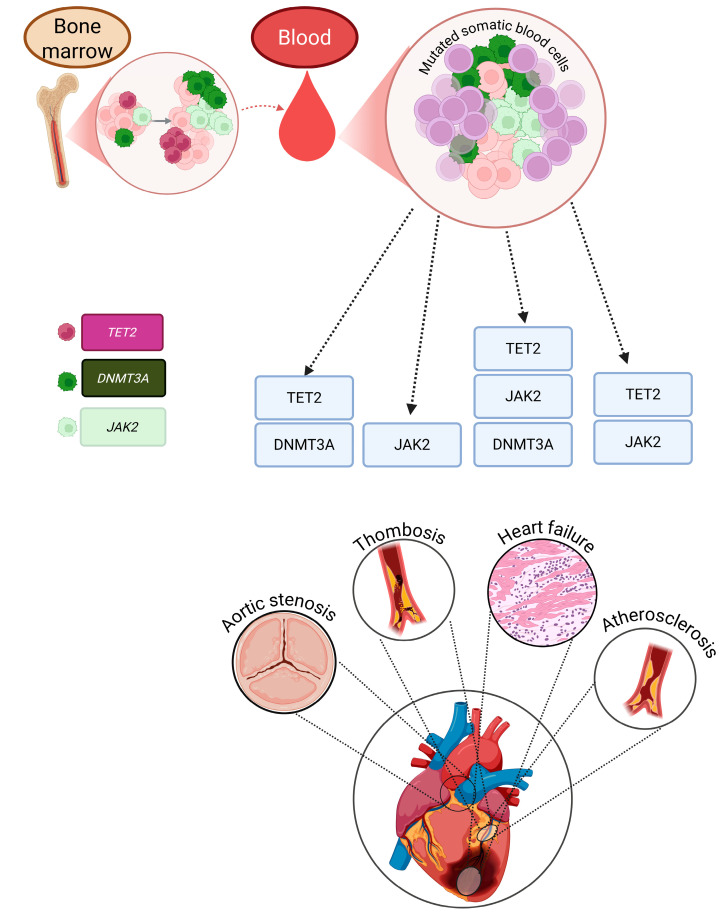
Major mutations and pathophysiological events driving and CVD in CHIP. These mutations increase the risk of cardiovascular entities like aortic stenosis, thrombosis, HF and atherosclerosis. Abbreviations: *DNMT3A*: DNA (cytosine-5)-methyltransferase 3A; *JAK2*: Janus kinase 2; *TET2*: ten-eleven translocation 2. Created in BioRender. Anastasiou, I. (2025) https://BioRender.com/92m4rc4 (accessed on 3 October 2025).

Despite important mechanistic evidence from murine and cellular studies demonstrating that mutant myeloid clones amplify IL-1β, IL-6, and TNF-α signaling, human data rely almost exclusively on single time point sequencing. Longitudinal human studies incorporating repeated CHIP assessments, VAF trajectories, and inflammatory biomarker profiling are urgently needed to clarify whether cytokine activation accelerates clonal expansion, whether expanding clones initiate systemic inflammation, or whether these processes evolve synergistically over time. Such temporal analyses would greatly strengthen causal inference regarding CHIP-associated cardiometabolic disease. Figure 2 illustrates the core inflammaging pathways linking CHIP, cytokine activation, and vascular/metabolic dysfunction, while Figure 3 depicts the major mutations and key pathophysiological events driving atherosclerosis and CVD in CHIP carriers.

## 5. CHIP and Metabolic Disorders

### 5.1. CHIP and Type 2 Diabetes

Recent research suggests that CHIP is more than a bystander in T2D, potentially representing a novel, independent risk factor for T2D and its microvascular complications, with potential implications for both prevention and management strategies [87]. Recently, Tobias et al. demonstrated that CHIP mutations in genes already implicated in CAD are also associated with T2D, pointing to shared aging-related mechanisms. Overall, CHIP carriers exhibited a 23% higher risk of developing T2D compared to non-carriers, with *TET2* and *ASXL1* mutations accounting for much of this effect [88].

Experimental studies have begun to elucidate the underlying mechanisms, showing that *TET2* deficiency enhances IR, a process linked to elevated IL-1β levels in white adipose tissue. Notably, this deleterious effect can be attenuated by inhibiting NLRP3 inflammasome-dependent IL-1β production [41]. Although direct metabolic defects in *TET2*-deficient macrophages have not been fully delineated, emerging evidence shows that *TET2* deficiency promotes mitochondrial stress and aberrant innate immune signaling, including the activation of the cGAS–STING and NLRP3 pathways, which secondarily reshapes macrophage metabolic programs. These inflammation-driven metabolic shifts may impair adipose-tissue homeostasis and contribute to IR [46,89,90].

Beyond disease onset, CHIP may also influence the progression of diabetic microvascular complications (DMCs). In a cohort of over 20,000 T2D patients initially free of DMCs or hematologic malignancies, the presence of CHIP predicted a 23% higher risk of developing DMCs over 13 years. The risk was especially pronounced for diabetic retinopathy (34%) and kidney disease (26%), whereas diabetic peripheral neuropathy (DPN) showed no overall association. Gene-specific analyses indicated that certain driver genes, including *DNMT3A*, *TET2*, *neurofibromin 1* (*NF1*), and spliceosome genes, were linked to the development of DMCs [91]. Additional findings revealed that CHIP prevalence was higher among patients without DPN compared to those with DPN (19.9% vs. 8.8%; *p* = 0.013). Specifically, carriers of DNMT3A mutations were less likely to exhibit neuropathic abnormalities, whereas *TET2* mutations were associated with impaired electrochemical skin conductance in the feet [92].

The impact of CHIP on T2D may be particularly pronounced in individuals with elevated concentrations of highly atherogenic low-density lipoprotein cholesterol (LDL-C). This suggests a gene–environment interaction in which pro-inflammatory myeloid clones amplify lipid-driven vascular injury, thereby accelerating the transition from metabolic dysfunction to overt cardiometabolic disease. Dyslipidemia is therefore an important modifier of CHIP-associated metabolic and inflammatory risk [93]. The clinical relevance of this interaction is illustrated in a prospective cohort of 1396 subjects with acute coronary syndrome (ACS) undergoing primary percutaneous coronary intervention (PCI). Among individuals with elevated atherogenic index of plasma (AIP), CHIP carriers experienced sharply increased all-cause mortality, with *TET2* mutations conferring an HR of 5.20 (95% CI: 1.75–15.41; *p* = 0.003), and *TET2/ASXL1* co-mutations an HR of 5.50 (95% CI: 2.02–13.15; *p* = 0.001). Therefore, CHIP may modify cardiovascular prognosis in the presence of dyslipidemia [94].

Taken together, these findings suggest that integrating CHIP genotyping with lipidomic profiles and inflammatory biomarkers may refine T2D risk stratification and identify individuals who could benefit from targeted anti-inflammatory or metabolic interventions.

### 5.2. CHIP and Chronic Liver Disease

Chronic liver disease (CLD) has become a major global health burden, ranking among the leading causes of mortality worldwide. Although it can arise from diverse etiologies, its clinical course typically converges on a common pathological sequence, marked by sustained inflammation, progressive liver injury, and the eventual development of fibrosis. Among its contributors, metabolic dysfunction-associated steatotic liver disease (MASLD), previously known as nonalcoholic fatty liver disease (NAFLD), accounts for more than half of all CLD cases. The hallmark of MASLD is the excessive accumulation of lipids within hepatocytes, a process closely intertwined with obesity, T2D, and IR [95,96].

Evidence linking CHIP to CLD has recently gained momentum. Recent genomic analyses across 214,563 individuals from four large cohorts (Framingham Heart Study, Atherosclerosis Risk in Communities Study, UK Biobank, and Mass General Brigham Biobank), demonstrated that CHIP was associated with a twofold higher risk of both prevalent and incident CLD (*p* < 0.001), as well as imaging-detected hepatic inflammation and fibrosis. CHIP appeared to accelerate liver injury primarily through dysregulated inflammatory responses. This effect was mediated by the activation of the NLRP3 inflammasome and the increased secretion of downstream pro-inflammatory cytokines by *TET2*-deficient macrophages [29]. Experimental data from the study show that mice transplanted with *TET2*-deficient hematopoietic cells develop more severe liver inflammation and fibrosis, mechanistically linked to NLRP3 activation and elevated cytokine expression. These inflammatory signals enhance hepatic immune activation and promote hepatic stellate cell activation and extracellular matrix deposition, thereby accelerating fibrosis progression in MASLD and steatohepatitis [29]. CHIP has also been independently associated with MASLD-related hepatocellular carcinoma (HCC), with mutations in non-*DNMT3A* drivers, notably *TET2* and *TP53*, showing the strongest associations [97]. Collectively, these observations highlight CHIP not only as a contributor to CLD progression but also as a potential biomarker for refining MASLD risk stratification, especially in patients with advanced fibrosis or increased cancer susceptibility. From a clinical standpoint, integrating CHIP screening with non-invasive fibrosis scores and metabolic risk markers may improve identification of MASLD patients at higher risk for progression, thereby informing surveillance strategies and early therapeutic intervention.

### 5.3. Could Obesity Exacerbate CHIP and Vice Versa?

Obesity is a major driver of chronic disease and represents a significant global public health challenge, with a broad impact on overall health and longevity. Beyond its well-established role in metabolic and cardiovascular disorders, including hypertension and T2D, obesity is strongly linked to at least 13 types of cancer, encompassing both solid tumors and hematologic malignancies, including preleukemic conditions such as MDS. Moreover, excess adiposity can exacerbate autoimmune and neurodegenerative diseases as well as outcomes in severe infections, such as COVID-19 [98,99,100,101,102,103,104,105,106].

While CHIP has emerged as a recognized risk factor for hematologic malignancies and CVD, only a small handful of studies have directly investigated its relationship with obesity [5]. Recent investigations have shed light on the intricate links between obesity, inflammation, CH, and their collective impact on CVD and cancer development. For example, Pasupuleti et al. demonstrated that metabolic and genetic factors act synergistically, with obesity-induced inflammation cooperating with CHIP-associated mutations, such as *TET2*, to accelerate both the onset of leukemogenesis and atherosclerotic CVD [30]. These findings suggest that obesity may not only exacerbate CHIP-related outcomes but also promote clonal expansion via inflammatory and metabolic stress.

Dietary factors are increasingly recognized as critical modulators of metabolic health. Zemel et al. reported that dietary calcium influences adiposity regulation and obesity risk, highlighting the role of nutrient intake in shaping both inflammatory pathways and fat deposition. Concurrently, individuals with obesity exhibit elevated levels of pro-inflammatory cytokines, including IL-6, as well as adipokines with pro-inflammatory activity, such as resistin, which contribute to IR and sustain a chronic inflammatory milieu that predisposes to chronic disease [107,108]. Pharmacological interventions may also intersect with these pathways. Among them, nifedipine has been investigated for its effects on cellular metabolism, demonstrating modulation of chondrocyte and stem cell metabolic activity [109]. Additionally, the drug has been shown to facilitate proliferation and migration of breast cancer cells, suggesting potential crosstalk between cardiovascular therapeutics and oncogenic pathways, while raising concerns about unintended consequences in individuals with both CHIP and obesity [110]. Lifestyle factors further modulate disease risk. For instance, smoking has been linked to *ASXL1* mutations associated with CHIP, whereas adherence to healthy lifestyle behaviors can mitigate some of these risks [111,112].

Epigenetic mechanisms play a central role in the development of obesity and metabolic dysregulation. Alterations such as *DNMT3A* dysfunction contribute to IR in adipose tissue and abnormal adipogenesis, reshaping gene expression patterns to favor excessive fat storage and increased inflammation [113,114]. However, the impact of obesity extends beyond metabolic disorders. It increases cancer risk by creating a pro-tumorigenic microenvironment fueled by chronic inflammation and altered immune responses, including the activity of tumor-associated macrophages [115,116]. Obesity-related inflammation not only promotes carcinogenesis but may also promote metastasis. These inflammatory and immune pathways also intersect with other conditions, including atrial fibrillation, atherosclerosis, and thrombosis, which share common mechanistic underpinnings centered on inflammatory mediators [117].

**Figure 4:**
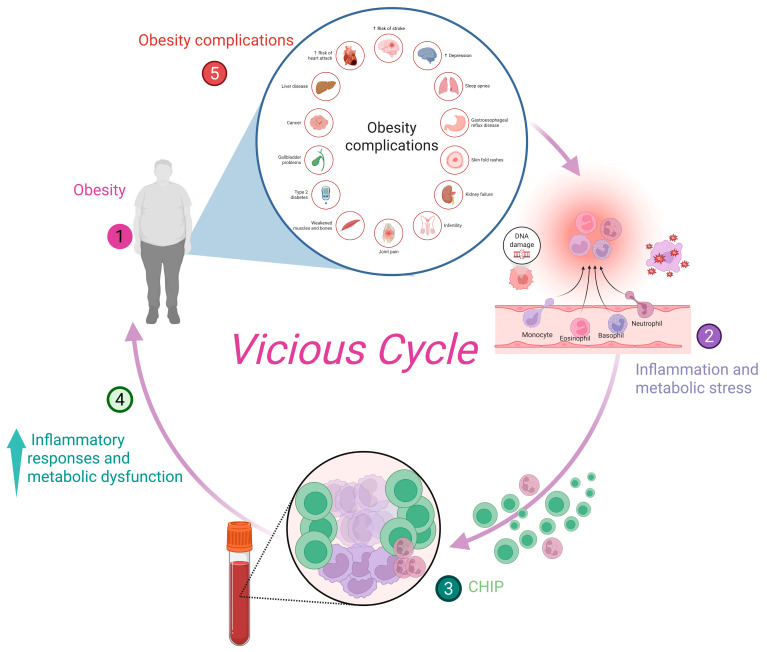
Hypothetical model illustrating the bidirectional relationship between obesity and CHIP. Obesity promotes CHIP clonal expansion through systemic inflammation and metabolic stress, while CHIP exacerbates obesity-related complications via enhanced inflammatory responses and metabolic dysfunction, creating a self-reinforcing cycle. Abbreviations: CHIP: clonal hematopoiesis of indeterminate potential; DNA (cytosine-5)-methyltransferase 3A; Created in BioRender. Anastasiou, I. (2025) https://BioRender.com/c00mfbd (accessed on 3 October 2025).

Overall, these findings suggest that moderating obesity-driven inflammatory pathways, particularly IL-6 signaling and NLRP3 inflammasome activation, may reduce clonal expansion and mitigate the metabolic sequelae associated with CHIP. Such strategies could offer novel opportunities for preventing or attenuating CHIP-related cardiometabolic disease in individuals with obesity. This concept is illustrated in Figure 4, which depicts the bidirectional interplay between obesity and CHIP, where systemic inflammation and metabolic stress foster the expansion of mutant hematopoietic clones, while CHIP-associated inflammatory activity further exacerbates obesity-related metabolic dysfunction, establishing a self-reinforcing vicious cycle.

## 6. CHIP and Other Cardiovascular-Related Disorders

### 6.1. CHIP and Chronic Kidney Disease

Although direct clinical investigations are limited, population-based studies suggest that CHIP may exacerbate renal injury, implicating hematopoietic somatic mutations in the development and progression of chronic kidney disease (CKD). A meta-analysis of 12,004 individuals without pre-existing renal disease demonstrated an age-dependent rise in CHIP prevalence, reaching 12.2% among participants over 70 years, and revealed that CHIP carriers had a significantly greater risk of kidney function decline, defined as a ≥30% reduction in estimated glomerular filtration rate (eGFR), with a 17% increased risk (95% CI, 1–36%) after adjustment for age, sex, baseline eGFR, urine albumin-to-creatinine ratio (UACR), and diabetes [118]. Findings from the UK Biobank reinforced these observations, showing that CHIP, particularly of myeloid lineage, was associated with an increased risk of CKD, with Mendelian randomization suggesting a possible causal relationship (*p* = 0.03). Importantly, variants other than *DNMT3A* were linked to more rapid CKD progression [119]. Supporting experimental evidence comes from a *TET2*-CH mouse model, in which a subpressor dose of angiotensin II leads to expansion of *TET2*-deficient myeloid clones, enhanced renal C-C motif ligand 5 (CCL5) chemokine expression and macrophage infiltration into the kidney. This is accompanied by renal NLRP3 inflammasome activation, elevated IL-1β and IL-18 levels, sodium retention and increased activation of the thiazide- and loop-sensitive transporters NCC and NKCC2; all of these changes are reversed by pharmacologic NLRP3 inhibition [120]. These findings provide a mechanistic link between CH, renal immune–inflammatory activation, altered tubular sodium handling and hypertension, which may in turn accelerate CKD progression [120]. Given that hypertension represents one of the most common causes of CKD, this pathway may have significant clinical implications and highlights the potential of immune-modulating interventions in individuals with *TET2*-CH [120,121]. On the other hand, in patients with T2D, CHIP did not correlate with accelerated renal functional decline [122]. Lastly, recent evidence also indicates that CHIP may predispose to acute kidney injury (AKI). Specifically, CHIP appears to impair renal recovery following AKI through maladaptive macrophage-mediated inflammatory responses. The association was strongest among patients with dialysis-requiring AKI and in carriers of non-*DNMT3A* variants, including mutations in *TET2* and *JAK2* [123].

### 6.2. CHIP and Gout

Gout, driven by monosodium urate (MSU) crystal deposition, has recently been linked to CHIP through shared inflammatory pathways. Acute flares of gout are marked by IL-1β release, and CHIP has similarly been associated with enhanced IL-1β signaling. This central concept provided the framework for the study by Agrawal et al., which leveraged large-scale Biobank datasets and currently represents the foremost evidence implicating CHIP in gout. This study demonstrated that individuals with CHIP exhibited both higher prevalence and increased incidence of gout compared with non-carriers, with the association being particularly pronounced among those harboring larger hematopoietic clones. Experimental evidence corroborated these observations. Hematopoietic cells deficient in *TET2* exhibited exaggerated IL-1β responses upon exposure to MSU crystals, an effect that was attenuated by pharmacologic inhibition of the NLRP3 inflammasome. Collectively, these data indicate that CHIP, especially TET2-driven, potentiates crystal-induced inflammation, thereby increasing susceptibility to gout and potentially exacerbating its clinical severity [47,124].

### 6.3. CHIP and Chronic Obstructive Pulmonary Disease

COPD is a progressive respiratory disorder defined by irreversible airflow limitation that arises primarily from prolonged exposure to noxious particles or gases, most notably tobacco smoke. Ongoing inflammatory activity in both the airways and parenchyma underpins its pathogenesis, ultimately resulting in structural alterations and lasting tissue harm. The prevalence and severity of COPD increase with advancing age, reflecting both cumulative exposure to deleterious environmental factors and the physiologic decline in pulmonary reserve [125,126].

Although current data remain scarce, recent research points toward a potential role of CHIP-associated epigenetic alterations in modulating COPD trajectories. In a cohort of 125 individuals with COPD evaluated by deep-targeted amplicon sequencing, somatic CHIP mutations were identified in approximately 20% of cases [127]. Notably, mutations in *DNMT3A* were the most frequent and were associated with hypomethylation of Phospholipase D Family Member 5 (*PLD5*) [128]. In DNMT3A-CHIP COPD, *PLD5* hypomethylation correlates with increased glycerophosphocholine, elevated IL-6/TNF-α and impaired lung function [128]. Given that phospholipase-D activity generates phosphatidic acid and lysophosphatidic acid, which are lipids that drive inflammatory mediator production, *PLD5* hypomethylation offers a plausible mechanism for the increased inflammatory state observed in these patients [128].

CHIP is more frequently observed in current COPD smokers, particularly those with a high smoking burden and more severe symptoms, both of which show significant associations with CHIP positivity. Furthermore, CHIP has been proposed as a potential prognostic marker in COPD, as evidence indicates that its presence is strongly linked to a higher risk of mild and overall acute exacerbations. Interestingly, mutations involving *ASXL1* were linked to a comparatively lower risk of total exacerbation events (adjusted OR 0.19). Conversely, its prognostic value may be limited in predicting progressive functional impairment functional deterioration [129]. These observations align with broader CHIP-associated inflammatory mechanisms described in cardiovascular and metabolic tissues. In the lung, mutant myeloid clones may similarly potentiate NLRP3 inflammasome activation in alveolar macrophages, promoting IL-1β-driven airway inflammation, impairing mucociliary clearance and increasing susceptibility to exacerbations [127,130]. This mechanistic overlap underscores the role of CHIP as a systemic amplifier of sterile inflammation across organ systems.

### 6.4. CHIP and Infectious Diseases

Recent evidence highlights a potential role of CHIP in shaping susceptibility and outcomes of infectious diseases with high cardiovascular burden, including COVID-19 and HIV. In a study of 90 hospitalized patients with COVID-19, mutations in CHIP-associated driver genes were present in 37.8% of cases, a prevalence more than double the 17% expected for individuals of similar age. Although CHIP carriers were more likely to require hospitalization, the presence of CHIP did not independently predict prognosis once admitted. Distinct immune alterations were noted across age groups. Individuals aged 45–65 with CHIP experienced sustained lymphopenia, whereas those over 65 demonstrated persistent neutrophilia. While a direct causal relationship between CHIP and elevated cardiac biomarkers could not be firmly established, the data point toward a possible link, supporting the hypothesis that CHIP may worsen COVID-19 severity through immune dysregulation and could serve as a marker for personalized risk assessment [131]. Concurrently, research in people living with HIV (PLWH) indicates nearly a twofold higher prevalence of CHIP compared with matched controls (*p* = 0.005), with ASXL1 emerging as the most frequent mutation. Given its known association with hematologic malignancies and CVD, CHIP may help explain the disproportionate cardiovascular burden in PLWH [132].

Table 3 summarizes clinical evidence linking CHIP to cardiometabolic and cardiovascular-related disorders, including infectious diseases with a high cardiovascular burden.

## 7. Screening and Diagnostic Perspectives

### 7.1. Molecular and Epigenetic Characterization of CHIP

In the TOPMed cohort of 4370 individuals with CHIP, mutational patterns were accompanied by distinct epigenetic and proteomic signatures that fell into several mechanistic themes. First, epigenetic remodeling was reflected by strong associations between CHIP and biological aging markers, such as accelerated PhenoAge, GrimAge, and HannumAge, particularly in carriers of *DNMT3A* and *TET2* mutations. Second, metabolic signaling pathways emerged prominently, with proteomic analyses identifying regulators of mitochondrial function, intermediary metabolism and energy homeostasis, including tissue Inhibitor of Metalloproteinases-1 (TIMP1), Glycine N-Methyltransferase (GNMT) and Anti-Müllerian Hormone (AMH), which correlated with clonal expansion rates. Third, coagulation and vascular remodeling features were evident, particularly among *TET2*-mutant carriers, who displayed signatures linked to endothelial activation, platelet function, and epithelial–mesenchymal transition (EMT). Collectively, these thematic clusters support the concept that CHIP shapes systemic biology through coordinated epigenetic, metabolic and vascular signaling programs, providing a foundation for future biomarker development [134].

### 7.2. Clinical Implementation and Screening Programs

Despite these molecular insights, integration of CHIP screening into routine practice remains limited, with evidence largely retrospective. However, observational studies have advanced the understanding of CHIP’s natural history, progression, and associations with hematologic and non-hematologic disease. These insights have prompted structured testing protocols at specialized centers. For example, the Mayo Clinic has established a research-based CHIP detection program (NCT#02958462), applying a targeted NGS panel of ~200 genes to peripheral blood while systematically excluding germline variants via parallel tissue analysis. Such programs exemplify how molecular research is shaping structured screening strategies, bridging biomarker discovery and clinical use.

### 7.3. Hematologic Screening: Cytopenias and Myeloid Neoplasms

Retrospective studies demonstrate that mutation testing in peripheral blood granulocytes improves diagnosis in unexplained cytopenias and enhances overall diagnostic accuracy for MNs. Approximately 45–60% of individuals with unexplained cytopenias, classified as CCUS, harbor CH mutations. High-risk features, including VAF ≥ 10%, multiple mutations, splicing factor gene mutations, or concurrent mutations in *DNMT3A*, *TET2*, or *ASXL1*, confer a positive predictive value of 0.8–1.0 for progression to MN [135]. Larger clones reflect greater proliferative fitness and sustained inflammatory signaling, while multiple mutations often indicate cooperative biological effects that impair differentiation, increase DNA damage tolerance and promote clonal dominance. These features collectively strengthen the predictive accuracy of mutation-based risk models. *TP53* and *SF3B1* mutations are particularly enriched in patients with anemia [136]. Elevated red blood cell distribution width (RDW) and leukocytosis are additional hematologic markers linked to increased CH likelihood. While prospective evidence for early intervention is lacking, identification of these mutations informs prognosis and follow-up strategies, making routine screening increasingly advocated in this setting [137,138].

CH-associated variants are also frequently identified incidentally during genomic testing for solid tumors such as lung or breast cancer [139]. Confirmatory testing in peripheral blood is essential due to overlap between CH-associated mutations and solid tumor drivers (*TP53*, *KRAS*, *EZH2*) [140,141]. Circulating tumor DNA (ctDNA) assays used for residual disease monitoring can yield false positives attributable to CH in ~10% of cases, necessitating careful interpretation [142]. Recognition of CH in this context is critical, as it can affect treatment decisions, risk assessment prior to adjuvant therapy, and differentiation of true disease progression from CH-related alterations.

### 7.4. Hematopoietic Stem Cell Transplantation and Cellular Therapy

Screening before autologous hematopoietic stem cell transplantation (auto-HCT) is advised, particularly in individuals >50 years or with prior chemoradiation exposure, as CH is associated with inferior overall and progression-free survival [143,144,145]. In multiple myeloma, lenalidomide, but not pomalidomide, promotes expansion of TP53-mutant clones, potentially influencing therapy [146]. Preliminary evidence in CAR-T therapy suggests pre-existing CH may reduce response, increase risk of high-grade cytokine release syndrome (CRS), and elevate incidence of therapy-related t-MN [147,148]. Research-based CH screening is recommended in this context, especially for older patients and those with prior cytotoxic therapy. Screening for *TP53* and *PPM1D* mutations is also suggested prior to peptide receptor radionuclide therapy (PRRT), adjuvant chemotherapy, or Poly(ADP-ribose)polymerase (PARP) inhibitor therapy, given their strong association with t-MN [149]. Detection of CH is not an absolute contraindication but requires a nuanced risk–benefit analysis, including consideration of alternative therapies.

### 7.5. Genetic Predisposition and Inherited Syndromes

Selective screening is recommended in patients with inherited bone marrow failure syndromes (IBMFS) or germline predisposition syndromes, especially when planning complex interventions like allogeneic HCT. Detection of CH informs treatment planning, risk stratification, and surveillance. Germline mosaic variants may confound interpretation, highlighting the importance of careful genetic counseling to distinguish somatic from inherited alterations. Early detection enables tailored management, reduces complications, and improves clinical outcomes [150].

### 7.6. Cardiovascular Screening

Evidence indicates that CH plays a causal role in atherosclerosis, with a hazard ratio of ~2.0, comparable to traditional risk factors such as hypertension (HR ~2.3) and T2D (HR ~3.1) [53]. The increased risk is independent of other conventional risk factors and is supported by both epidemiological and experimental data, including murine models showing that CHIP mutations causally accelerate atherosclerosis [53,151]. In individuals with atherosclerotic vascular disease, particularly those without conventional risk factors, systematic CH screening, including cytokine profiling, could refine risk stratification and guide management.

## 8. Therapeutic Perspectives in the Context of Inflammaging

### 8.1. Interventional Evidence and Clinical Trials

Interventional evidence at the CHIP–cardiometabolic interface remains nascent and largely focused on inflammaging pathways, with the most mature targets involving the IL-1β/IL-6–NLRP3 axis. Among existing studies, only one trial (NCT0609766) prospectively enriches for CHIP in CAD (*DNMT3A/TET2*, VAF ≥ 2%) and evaluates oral NLRP3 inhibition and IL-1 family blockade, yielding short-term biomarker readouts (IL-6, IL-18, hsCRP) rather than hard clinical endpoints. Although the first CHIP-enriched interventional study, the NCT06097663 trial is hypothesis-generating and underpowered for clinical endpoints. Its short duration and reliance on biomarker readouts highlight the necessity for adequately powered, VAF-stratified, mutation-specific randomized trials capable of evaluating hard cardiovascular outcomes. However, most CHIP-focused interventional studies to date, including NCT06097663, are designed primarily to assess safety, feasibility and biomarker modulation rather than clinical event reduction, and are not powered to detect differences in major adverse cardiovascular outcomes [152]. Interestingly, an exploratory analysis of the CANTOS trial reinforced earlier evidence linking CHIP to an increased risk of cardiovascular events and suggested that patients harboring *TET2* mutations may derive greater benefit from canakinumab therapy than those without CHIP. Specifically, among individuals with *TET2*-driven CHIP, treatment with canakinumab was associated with a HR for MACEs of 0.38 (95% CI, 0.15–0.96) compared with placebo, representing a numerically larger treatment effect than that observed in the overall trial population. In contrast, no similar benefit was observed in carriers of *DNMT3A* mutations or in participants without CHIP [21].

Very recently, another exploratory analysis of the LoDoCo2 (Low-Dose Colchicine 2) trial investigated whether assignment to colchicine, compared with placebo, influenced CH expansion in individuals with chronic CAD. It also evaluated the relationship between colchicine use and longitudinal changes in inflammatory biomarkers according to CH status. Participants assigned to placebo demonstrated a 14.9% annual increase in CH clone size (β_time_ = 0.14; 95%, 95% CI: 0.08 to 0.21), whereas those treated with colchicine exhibited a smaller, non-significant increase of 6.3% per year (β_time_ = 0.06; 95% CI: −0.01 to 0.14). Nevertheless, the overall difference between treatment groups did not reach statistical significance (*p* for interaction = 0.13). Moreover, compared with placebo, colchicine use was associated with reduced clonal growth in *TET2*-driven CH (β_time_ on colchicine: 0.09 [95% CI: −0.04 to 0.22] vs. β_time_ placebo: 0.27 [95% CI: 0.16 to 0.37]; *p* for interaction = 0.04). Notably, among individuals with non-*DNMT3A*-driven CH, IL-6 levels increased to a lesser extent in those receiving colchicine vs. placebo over 1 year (30.0% vs. 98.1% increase, respectively; *p* for interaction = 0.01). Collectively, these findings support that low-dose colchicine may limit clonal expansion and inflammatory activity in specific CH subtypes, particularly those driven by TET2 mutations [153].

IL-6-targeted trials (e.g., ziltivekimab) have demonstrated robust inflammatory biomarker reductions in high-risk cardiometabolic populations but lack CHIP enrichment and definitive clinical outcome data [154,155] (NCT06118281). Repurposed agents aligned with the inflammaging paradigm, such as statins (atorvastatin and rosuvastatin), metformin, and colchicine, have mechanistic plausibility via lipid, mitochondrial, and inflammasome modulation, with supportive signals from hematologic or cardiovascular studies; however, CHIP-stratified interventional evidence remains limited or indirect [87,156,157] (NCT05483010) Ongoing work in CCUS/MDS (e.g., statins) informs feasibility more than cardiometabolic outcomes. Notably absent are genotype-specific cardiovascular trials for *JAK2* (thrombo-inflammation) or systematic strategies keyed to clone size (VAF) and multi-mutation risk.

Table 4 highlights a field transitioning from association studies to precision interventions, though current trials are small, heterogeneous, and largely biomarker-centric. The next step is genotype- and VAF-enriched, outcome-powered studies that validate surrogate markers and test mechanism-matched therapies within the broader framework of inflammaging. Taken together, the consistent therapeutic signals observed across IL-1β blockade, IL-6 inhibition, and emerging inflammasome-targeted agents support a causal inflammatory loop linking CHIP-driven myeloid activation to atherogenesis and cardiometabolic injury.

### 8.2. Inflammation and Immune Mechanisms

No standardized criteria exist to identify individuals who would most benefit from CHIP-targeted therapies. Nonetheless, preclinical studies provide promising directions. In mouse models, TET2 deficiency in bone marrow accelerates atherosclerosis. Macrophages lacking TET2 produce excess IL-1β via NLRP3 inflammasome activation, promoting vascular inflammation and plaque formation. Pharmacologic inhibition of NLRP3 in these mice reduced plaque size and endothelial adhesion molecule expression, supporting inflammasome-targeted therapies as potential protective strategies against CHIP-driven atherosclerosis [40]. While murine *TET2*- and *JAK2*-mutant models provide sufficient causal evidence, e.g., *TET2* deficiency increases aortic plaque area by ~40–60% in LDLR−/− mice and *JAK2V617F* expression increases thrombosis susceptibility [40,160] human studies remain associative [11,161]. The absence of interventional data linking clone suppression to clinical risk reduction underscores a translational gap that future genotype-enriched trials must address.

Cavalli and Dinarello have emphasized the therapeutic potential of IL-1 blockade for inflammatory diseases, noting that agents such as anakinra and canakinumab reduce inflammation and improve clinical outcomes [162]. Building on this, Svensson et al. explored canakinumab in *TET2*-driven CH carriers in an exploratory analysis of CANTOS. Individuals with *TET2* mutations showed greater reduction in cardiovascular events following IL-1β blockade, supporting the concept that CHIP-associated, *TET2*-driven inflammation can be mitigated to lower cardiovascular risk [21].

CHIP mutations in genes like *TET2* and *DNMT3A* impair epigenetic regulation, increasing inflammation. DNA damage, including mitochondrial DNA release, activates the STING pathway, triggering type I IFN responses and chronic inflammation. Dysregulated DNA damage responses and STING activation in CHIP exacerbate disease progression and elevate hematologic malignancy risk, suggesting that targeting DNA damage response or STING signaling may offer novel therapeutic avenues [46]. Neutrophils also play a dual role in CVD, contributing to both inflammation and resolution. NETs promote thrombosis and plaque destabilization in cardiovascular disorders. Elevated NET formation correlates with higher thrombotic risk. Neutrophil-mediated cytokine secretion and interactions with other immune cells amplify vascular inflammation [163,164]. The JAK-STAT pathway mediates cytokine-driven neutrophil activation, representing a therapeutic target for CVD associated with systemic inflammation. Inhibition of JAK-STAT signaling may reduce NETosis, thrombosis, and vascular injury [165].

### 8.3. Epigenetics, Metabolism, and Clonal Expansion

Vitamin C (ascorbate) is critical for hematopoietic stem cell function and epigenetic regulation, enhancing TET enzyme activity and DNA demethylation. Restoration of TET2 function via vitamin C inhibits leukemia progression and maintains normal stem cell differentiation [166,167]. Although vitamin C trials exist (NCT03418038; NCT03682029), these focus on hematologic endpoints in CCUS/low-risk MDS/CMML, but not in cardiometabolic outcomes [168]. On the other hand, preclinical and human data suggest that a pro-inflammatory bone marrow microenvironment can favor the expansion of CHIP clones. Experimental models show that mutant *TET2*, *DNMT3A*, and *JAK2* clones can gain a proliferative advantage that may be attenuated by interventions targeting mitochondrial metabolism (e.g., metformin) or IL-1/NLRP3 signaling, supporting mechanism-based drug repurposing strategies that still require formal clinical testing [169].

## 9. Challenges and Controversies

Despite significant progress in linking CHIP to cardiometabolic risk, several challenges and controversies continue to hinder its translation into clinical practice. A major limitation is the lack of consistency in how CHIP is defined and assessed across studies. Differences in operational definitions, sequencing depth, minimum VAF thresholds (often 2%, though clinically meaningful risk generally emerges at 5–10%), and the inclusion or exclusion of mosaic chromosomal alterations all shape prevalence estimates and risk associations. Heterogeneity in study populations adds another layer of variability, complicating attempts to draw broad conclusions [123]. Current prognostic models (e.g., CHRS, CCRS, MN-Predict) were developed to predict progression of CCUS and have not been validated for cardiovascular outcomes, limiting their relevance for cardiometabolic risk stratification [6,49,123].

The pathogenic and clinical significance of CHIP is highly mutation- and clone-specific. As previously discussed, *TET2* mutations are linked to increased inflammatory signaling, whereas *JAK* drives thrombosis through mechanisms including NET formation and endothelial activation. However, *DNMT3A* mutations in isolation frequently yield inconsistent associations with mortality or malignant transformation. This heterogeneity raises questions about whether all CHIP warrants intervention or whether clinical management should be restricted to high-risk subtypes [13,170]. The contribution of inflammation in CHIP-associated disease is well supported by experimental evidence; yet it remains uncertain whether pharmacological suppression in CHIP carriers leads to measurable improvements in hard clinical outcomes. Epidemiological studies indicate that CHIP is linked to the occurrence of first but not recurrent coronary events, suggesting that its prognostic value may be context dependent [10]. This has direct clinical consequences, since treating asymptomatic older adults with low-burden clones could turn a latent biological finding into a disease label, increasing anxiety and exposing patients to drug toxicities without proven therapeutic benefits [10].

Designing interventional trials in CHIP is inherently difficult, given the low annual progression rates (approximately 0.5–1% for hematologic malignancy and variable for CVD) and the long latency before clinical endpoints become apparent [15,171]. Proposed surrogate markers, including VAF, inflammatory cytokines and epigenetic clocks are appealing but remain unvalidated as predictors of clinically meaningful outcomes. Uncertainty also extends to trial eligibility, since it is unclear whether interventions should be limited to individuals with high VAF, specific driver mutations or concomitant cardiometabolic disease. Since most CHIP carriers are otherwise healthy older adults identified incidentally, exposing them to long-term pharmacologic therapy raises significant ethical concerns [15,172]. Attitudes among potential participants are heterogeneous; some may value proactive risk reduction, whereas others object to being categorized as “pre-diseased.” Safeguarding informed consent, preventing therapeutic misconception, and minimizing iatrogenic harm are therefore critical. Practical considerations also shape trial design. Oral agents with established safety profiles and secondary indications, such as statins, metformin, or colchicine are more likely to be acceptable, while intravenous therapies or expensive biologics such as canakinumab introduce substantial feasibility and cost-effectiveness barriers [173]. Moreover, screening strategies for CHIP remain contentious. Universal testing would identify a substantial number of carriers, but in the absence of established management pathways this approach risks generating a large diagnostic gray zone. Conversely, highly selective screening restricted to individuals with unexplained cytopenias, elevated inflammatory burden or premature CVD may overlook carriers who might derive benefit from earlier intervention. The trade-offs between sensitivity, specificity, cost, and downstream clinical utility are not yet resolved. These uncertainties highlight the importance of clinical trials that are staged, ethically defensible, and grounded in biological rationale. A central debate persists over whether CHIP should be regarded primarily as a biomarker of risk or as a direct therapeutic target. Until surrogate endpoints are validated and risk-adapted strategies are developed, enthusiasm for CHIP-directed interventions must be tempered by the potential for overdiagnosis, overtreatment and inequitable access [11]. Lastly, an important methodological limitation of the existing literature is that nearly all human studies assess CHIP at a single time point. This cross-sectional design restricts the ability to infer temporal relationships between clonal expansion, inflammatory activation, and the onset or progression of cardiometabolic disease. Because CHIP prevalence and clone size increase with aging, longitudinal studies with repeated sequencing will be essential to clarify whether mutation expansion precedes CVD, arises as a consequence of chronic inflammatory states or evolves in parallel with other aging-related processes. Such temporal analyses may also reveal whether CHIP serves as a broader amplifier of age-related atherosclerosis and inflammaging, extending its significance beyond discrete cardiovascular phenotypes [28].

## 10. Conclusions

**Figure 5:**
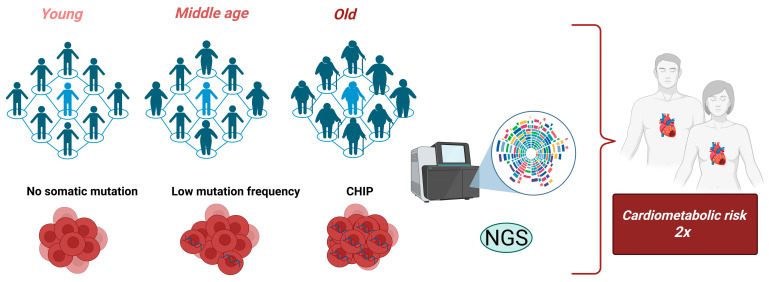
The CHIP continuum, showing how age-related somatic mutations progress from absence in youth to low mutation burden in middle age, and eventually to large clone sizes in older age. Larger clones are linked to elevated cardiometabolic risk and disease, highlighting the importance of longitudinal monitoring of clonal dynamics. Abbreviations: NGS: Next-generation sequencing; Created in BioRender. Anastasiou, I. (2025) https://BioRender.com/8mrc51p (accessed on 3 October 2025).

CHIP has evolved from a hematologic curiosity to a clinically meaningful driver of cardiometabolic risk, with meta-analyses linking it to higher all-cause mortality and increased coronary, HF, and stroke events, particularly with larger clone sizes. Pro-inflammatory pathways, including IL-1β/IL-6 signaling, NLRP3 inflammasome activation, and JAK2-mediated thrombo-inflammation, explain its role in atherosclerosis, metabolic dysfunction, and thrombotic risk, highlighting druggable targets for prevention. However, clinical risk depends on mutation type, clone size, co-mutations, and host factors, and current high-risk models require adaptation for cardiovascular endpoints. Biomarkers such as epigenetic clocks and proteomic signatures may complement sequencing for risk prediction and monitoring. Pragmatic screening strategies remain limited, with current implementation focused on research panels and retrospective data, emphasizing careful case selection and safeguards against false positives. While potential therapeutic targets exist, translating findings into effective interventions is challenging, especially given CHIP’s interactions with obesity, diabetes, and cancer. Ethical, patient-centered trial designs with validated endpoints are critical to avoid over-medicalization and ensure safety, feasibility, and generalizability. Figure 5 depicts the continuum of CHIP development across the lifespan, illustrating how somatic mutations in hematopoietic cells progress from no mutation in youth to low mutation frequency in middle age, and then to larger clone sizes in old age. This progression is associated with an increasing cardiometabolic risk and disease, highlighting the importance of monitoring clonal expansion over time.

## 11. Clinical Practice Points for Clinicians and Trialists

- Treat CHIP as a measurable cardiovascular risk factor, especially in carriers with high-VAF or high-risk genotypes, integrating inflammation markers to refine risk and follow-up.
- Prioritize enriched screening where clinical utility is highest (e.g., CCUS, unexplained cytopenias, incidentally discovered clonal variants during oncology testing), with confirmatory workflows to avoid ctDNA-related misclassification.
- Consider mechanism-matched prevention: IL-1β/IL-6/NLRP3-directed strategies for inflammatory clones; antithrombotic/thrombo-inflammatory approaches in JAK2; and metabolic/epigenetic modulators for cardiometabolic phenotypes.
- Build consent and monitoring around the asymptomatic nature of most carriers and select high-risk participants to balance benefit and risk, ensuring also inclusive enrollment.
- Align with the CCUS workflow where appropriate (e.g., staged intervention in higher-risk states), while recognizing progression risks differ between CHIP and CCUS.

### Future Research Agenda

- Risk stratification for CVD endpoints: Adapt and validate CHIP/CCUS risk scores for cardiovascular outcomes; prospectively test mutation- and VAF-based thresholds that trigger preventive therapy.
- Biomarker validation: Qualify surrogates that track risk and treatment response (VAF kinetics, hsCRP/IL-6, inflammasome readouts, epigenetic age acceleration, proteomic panels) against hard clinical outcomes.
- Precision interventional trials: Conduct CHIP-enriched randomized studies (e.g., TET2/DNMT3A for IL-1β/IL-6/NLRP3 strategies; JAK2 for thrombotic endpoints), with pre-specified genetic strata and clinically meaningful cardiovascular outcomes.
- Pragmatic and ethical design: Use oral/low-burden agents when possible; embed patient-reported outcomes and equity plans; ensure transparent communication that early-phase trials test surrogates and may not directly benefit participants.
- Implementation: Define when and where to screen (cardiologic, endocrine, oncology clinics), how to triage incidental findings, and how to handle ctDNA–CH discordance in multidisciplinary pathways.
- Interfaces with obesity and associated metabolic disorders: Prospectively test whether targeting inflammation/energy metabolism (e.g., NLRP3/IL-6 pathways, statins/metformin/colchicine) disrupts the obesity–CHIP feedback loop and improves cardiometabolic outcomes.

## Figures and Tables

**Table 1:** Some important in vitro, animal and human studies on CHIP and its association with cardiometabolic disease.

Level/Method	Model or Technique	Main Findings	Relevance	References
In vivo (mouse)	*TET2*-deficient hematopoietic cell models	Accelerated atherosclerosis; IL-1β/IL-6 via NLRP3; worsened IR	Demonstrates causal inflammatory mechanisms linking CHIP with atherosclerosis and metabolic dysfunction (IR and T2D)	[40,41]
In vivo (mouse)	Murine bone marrow transplantation (*TET2/DNMT3A*)	*TET2* or *DNMT3A*-deficient HSPCs increase cardiac hypertrophy, renal fibrosis and inflammation after angiotensin II infusion challenge.	Demonstrates causal link between CHIP mutations and cardiac dysfunction; gene-specific effects noted.	[42]
In vivo (mouse)	*DNMT3A* loss-of-function models	Loss of *DNMT3A* enhances macrophage inflammatory activity in vitro and generates in vivo a macrophage population with resident-like features and a pro-inflammatory cytokine signature, resembling the effects of *TET2* deficiency.	Shows that loss of either gene activates a shared innate immune pathway, offering a mechanistic explanation for the elevated atherosclerotic risk seen in carriers of these common CHIP mutations.	[43]
In vivo (mouse)	*JAK2V617F* myeloid expression	Increased NET formation; arterial and venous thrombosis; endothelial injury	Explains thrombotic complications of *JAK2*-mutant CHIP	[44]
In vivo (mouse)	*TET2*-deficient hematopoietic cells in non-alcoholic steatohepatitis	More severe liver inflammation and fibrosis, mediated by NLRP3 inflammasome activation and increased inflammatory cytokine expression in Tet2-deficient macrophages.	CHIP is linked to increased liver inflammation and accelerated chronic liver disease progression through dysregulated inflammatory signaling.	[29]
In vitro	*TET2*- or *DNMT3A*-deficient macrophages (murine, human monocyte-derived macrophages)	− *TET2*-deficient murine macrophages have increased expression of IL-1β and IL-6 following inflammatory stimulation, and human *TET2*-mutant macrophages from patients with MN also display elevated IL-6. − *DNMT3A*-deficient human macrophages exhibit a type I interferon response and increased inflammatory gene expression	Provide in vitro evidence that *TET2-* or *DNMT3A*-deficient macrophages have a cell-intrinsic inflammatory phenotype characterized by elevated cytokine production	[45,46]
In vitro	*TET2*-deficient murine macrophages exposed to MSU crystals	Increased IL-1β; reversed by NLRP3 inhibition	CHIP enhances NLRP3-dependent inflammatory activation triggered by MSU crystals in patients with gout	[47]
Diagnostic (NGS)	NGS of peripheral blood. The panel covered 54 genes, including CHIP-associated genes; quantified clone size.	NGS of 173 individuals over 75 years of age without prior hematologic disease revealed CH in 30.6%, predominantly involving *DNMT3A*, *TET2*, and *ASXL1* mutations.	Gold standard for CHIP diagnosis; informs risk stratification and clinical associations.	[48]
Prospective study/Diagnostic (CHRS tool)	− ARIC prospective cohort study of 3871 adults aged 67–90 years without hematologic malignancy across four U.S. centers, with samples collected in 2011–2013 and sequencing and analysis completed in 2022–2023. − CHRS scores, derived from 8 demographic, hematologic (blood count parameters), and molecular variables, classified individuals with CH into low- (≤9.5), intermediate- (>9.5 to <12.5), and high-risk (≥12.5) categories	24.2% had CH. Based on CHRS, 59.9% were low risk, 33.9% intermediate risk, and 6.2% high risk. Over 7.1 years of follow-up, mortality occurred in 19.4% without CH and 27.1% with CH. Mortality increased markedly across CHRS groups: 22.8% (low risk), 29.2% (intermediate risk), and 56.9% (high risk). Compared with no CH, only high-risk CH was significantly associated with all-cause mortality (sHR 2.52). In the high-risk group, risks of death from hematologic malignancy (sHR 25.58) and cardiovascular causes (sHR 2.91) were substantially elevated.	The CHRS, a practical tool based on routine clinical variables to estimate MN risk in CHIP/CCUS, was strongly associated with higher all-cause, hematologic and cardiovascular mortality.	[49]
Observational cohort study	DNA-methylation array and whole-genome sequencing data from 4 cohorts comprising 5522 individuals to study the association between CHIP, epigenetic clocks and health outcomes	CHIP carriers harboring multiple mutations showed the greatest increase in age acceleration. Individuals with CHIP and age acceleration presented a higher risk of mortality and CHD compared to individuals with only CHIP or age acceleration.	Support a link between CHIP, accelerated biological aging and the pro-inflammatory state characteristic of inflammaging.	[27]
Large-scale plasma proteomics analysis (largest-to-date characterization of the plasma proteome in CHIP)	Blood-based DNA sequencing and proteomic analysis using high-throughput proteomic platforms (SomaScan and Olink) from 61,833 participants (3881 with CHIP) from TOPMed and UK Biobank to identify proteins associated with CHIP and specific driver mutations.	Distinct proteomic signatures, including elevated levels of TIMP1, GNMT, AMH, MZF1 associated with the presence and type of CHIP mutation. These protein levels varied by mutation, sex, and race, and were enriched for pathways related to immune response and inflammation.	− Support the use of these plasma proteins as potential biomarkers for tracking clonal expansion and mutation-specific biological activity in CHIP. − CHIP is a systemic inflammatory driver that reshapes the circulating proteome toward IL-1β/NLRP3/IL-6-mediated pathways central to atherosclerosis and cardiometabolic disease. Gene-specific proteomic signatures (especially for *TET2*, *ASXL1*, *JAK2*) explain varying cardiovascular risks among CHIP carriers. CHIP shares a large inflammatory proteome with CAD and causally modifies inflammatory mediators.	[50]

Abbreviations: AMH: Anti-Müllerian Hormone; ARIC: Atherosclerosis Risk in Communities; ASXL1: Additional sex combs-like 1; CH: Clonal Hematopoiesis; CHD: Chronic Heart Disease; CHRS: Clonal Hematopoiesis Risk Score; DNMT3A: DNA (cytosine-5)-methyltransferase 3 alpha; GNMT: Glycine N-methyltransferase; IL-1β: Interleukin-1 beta; IL-6: Interleukin-6; IR: Insulin Resistance; JAK2: Janus kinase 2; MSU: Monosodium urate; MZF1: Myeloid zinc finger 1; NGS: Next-generation sequencing; NLRP3: NOD-like receptor family pyrin domain-containing 3; sHR: sub-distribution hazard ratio; T2D: type 2 diabetes mellitus; TET2: Ten-eleven translocation 2; TIMP1: Tissue inhibitor of metalloproteinases-1.

**Table 2:** Clinical associations of CHIP across different cardiovascular disease phenotypes.

Author, Year	CVD Phenotype	Study Design	Results	Conclusions
Heimlich et al., 2024, [54]	CAD	Observational cohort study involving 1142 patients undergoing coronary angiography	✓ CHIP mutations were identified in 18.4% of individuals undergoing coronary angiography ✓ CHIP was linked to a greater likelihood of significant left main artery (OR 2.44, 95% CI 1.40–4.27; *p* = 0.0018) and LAD artery (OR 1.59, 95% CI 1.12–2.24; *p* = 0.0092) obstructive coronary disease ✓ TET2 variant demonstrated the strongest association with left main coronary stenosis, exceeding the impact of other CHIP-related mutations	CHIP is associated with a distinct coronary phenotype, marked by increased risk of obstructive left main and LAD arteries stenosis, particularly in *TET2* mutation carriers
Yu et al., 2021, [57]	HF	Prospective cohort study of 56,597 individuals without prior HF or hematologic malignancy	✓ 6% of participants had CHIP, and 8.3% exhibited HF over up to 20 years of follow-up ✓ CHIP carriers had a 25% higher risk of developing HF (HR 1.25; 95% CI 1.13–1.38) ✓ *ASXL1*, *TET2*, and *JAK2* variants were linked to increased HF risk, while *DNMT3A* was not ✓ VAF > 10% conferred a greater HF risk (HR 1.29; 95% CI 1.15–1.44) ✓ ASXL1 variants were associated with lower left ventricular ejection fraction	CHIP, especially mutations in *ASXL1*, *TET2*, and *JAK2*, is an emerging risk factor for HF
Sikking et al., 2024, [62]	DCM	Observational cohort study including 520 patients with DCM	✓ CH driver mutations were found in 109 (21%) of patients (41% with VAF ≥ 2%, 31 with VAF < 0.5%) ✓ CH presence predicted higher risk of cardiac death (HR 2.33; 95% CI 1.24–4.40; VAF cutoff 0.36%) and all-cause mortality (HR 1.72; 95% CI 1.10–2.69; VAF cutoff 0.06%), independent of clinical covariates, including age, gender, LVEF, and NYHA functional classification	CH serves as an independent predictor of cardiac and all-cause mortality in DCM, regardless of clone size
Bhattacharya et al., 2022, [64]	Stroke	Prospective multi-cohort analysis including 78,752 participants from 8 cohorts and Biobanks	✓ CHIP carriers had a higher risk of total stroke (HR 1.14; 95% CI 1.03–1.27; *p* = 0.01) after adjusting for demographic factors, including age, gender, and race ✓ Hemorrhagic stroke risk was significantly increased (HR 1.24; 95% CI 1.01–1.51; *p* = 0.04), with additional links to small vessel ischemic stroke ✓ *TET2* variants showed the strongest correlation with both total and ischemic stroke, while *TET2* and *DNMT3A* were each linked to higher risk of hemorrhagic stroke	CHIP is linked to a higher risk of stroke, especially hemorrhagic and small vessel ischemic subtypes
Büttner et al., 2023, [70]	PAD	Pilot observational study including 31 patients with PAD undergoing open surgical procedures	✓ CH mutations were found in 14 of 31 patients (45%), with 5 patients carrying multiple mutations ✓ The most frequent mutations were *TET2* (55%) and *DNMT3A* (40%) ✓ 88% of blood-detectable mutations were also present in atherosclerotic plaques ✓ 12 patients had CH mutations in perivascular fat or subcutaneous tissue	CH may contribute to PAD development, as mutations are found in both blood and affected vascular tissues
Schuermans et al., 2024, [26]	Arrythmias	Observational cohort study including 410,702 UK Biobank participants without preexisting arrhythmias	✓ 3.4% had any CHIP and 2.2% had large CHIP ✓ Associations varied across arrhythmia subtypes and were strongest for cardiac arrest ✓ Any and large CHIP were correlated with statistically significant higher risks of supraventricular arrhythmias (HR 1.11 and 1.13), bradyarrhythmias (HR 1.09 and 1.13), and ventricular arrhythmias (HR 1.16 and 1.22), independent of CAD and HF ✓ Elevated arrhythmia risk for all CHIP driver genes apart from *DNMT3A* ✓ Large CHIP was linked to 1.31-fold higher odds of being in the top quintile of myocardial fibrosis measured by CMR (95% CI 1.07–1.59; *p* = 0.009)	CHIP may serve as a novel risk factor for incident arrhythmias, suggesting that targeting CHIP or its pathways could offer opportunities for prevention and treatment
Schuermans et al., 2025, [71]	Myocarditis/ Pericarditis	Observational population-based cohort study using data from 335,426 participants adults from the UK Biobank	✓ 3.3% had any CHIP, and 2.2% had large CHIP ✓ 0.11% developed myocarditis or pericarditis ✓ Participants with any CHIP had a 1.75-fold higher risk of developing myocarditis or pericarditis (95% CI, 1.14–2.68; *p* = 0.01), while those with large CHIP had a 2.07-fold higher risk (95% CI, 1.28–3.33; *p* = 0.003) ✓ DNMT3A mutations were associated with an increased risk of pericarditis (HR 2.22; 95% CI, 1.17–4.21; *p* = 0.01), and *TET2* mutations were linked to a higher risk of myocarditis (HR 3.65; 95% CI, 1.16–11.49; *p* = 0.03) ✓ CHIP showed stronger associations with myocarditis and pericarditis than with other CVDs ✓ Any CHIP was also linked to a 1.27-fold increased risk (95% CI 1.16–1.39; *p* < 0.001) of non-cardiac immune-mediated inflammatory diseases	CHIP is a significant risk factor for myocarditis and pericarditis in middle-aged individuals, suggesting that targeting CHIP or its downstream pathways could offer preventive and therapeutic potential

Abbreviations: 95% CI: 95% confidence interval; ASXL1: additional Sex Combs-Like 1; CAD: coronary artery disease; CHIP: clonal hematopoiesis of indeterminate potential; CMR: cardiac magnetic resonance; CVD: cardiovascular disease; DCM: dilated cardiomyopathy; DNMT3A: DNA (cytosine-5)-methyltransferase 3A; HF: heart failure; HR: hazard ratio; LAD: left anterior descending; JAK2: Janus kinase 2; LVEF: left ventricular ejection fraction; NYHA: New York Heart Association; OR: odds ratio; PAD: Peripheral arterial disease; TET2: ten-eleven translocation 2; VAF: variant allele frequency.

**Table 3:** Clinical correlations of CHIP with cardiometabolic and cardiovascular-related conditions.

Author, Year	Disorder	Study Design	Results	Conclusions
Tobias et al., 2023, [88]	T2D	Prospective cohort analysis including 17,637 participants from six cohorts, without prior T2D, CVD, or malignancy	✓ CHIP carriers had a 23% higher risk of developing T2D compared with non-CHIP carriers (HR 1.23; 95% CI 1.04–1.45) ✓ *TET2* mutations were linked to increased T2D risk (HR 1.48; 95% CI 1.05–2.08), while *ASXL1* mutations also showed elevated risk (HR 1.76; 95% CI 1.03–2.99) ✓ *DNMT3A* mutations were not significantly associated (HR 1.15; 95% CI 0.93–1.43)	CHIP is linked to a higher risk of T2D, with mutations in genes also associated with CHD and mortality, indicating a shared aging-related pathological pathway
Marchetti et al., 2024, [97]	MASLD-related HCC	Case–control study including 208 patients with MASLD-related HCC and 673 controls (414 with and 259 without advanced fibrosis)	✓ CHIP was identified in 116 participants (13.1%), most commonly in *DNMT3A*, *TET2*, *TP53*, and *ASXL1*, and was significantly associated with age (*p* < 0.0001) and advanced liver fibrosis (*p* = 0.001) ✓ Elevated AST levels predicted non-*DNMT3A* CHIP, particularly for clones with VAF ≥ 10% (OR 1.14 and 1.30; *p* < 0.05) ✓ After adjusting for sex, diabetes, and polygenic risk, CHIP was associated with cirrhosis (OR 2.00; 95% CI 1.30–3.15; *p* = 0.02) and with HCC, even after further adjustment for cirrhosis (OR 1.81; 95% CI 1.11–2.00; *p* = 0.002) ✓ Non-*DNMT3A CHIP* and *TET2* mutations remained independently associated with HCC after full adjustment for age and clinical/genetic covariates (OR 2.45; 95% CI 1.35–4.53 and OR 4.8; 95% CI 1.60–17.0; *p* = 0.02)	CHIP shows an independent association with MASLD-related HCC, driven mainly by non-*DNMT3A* and *TET2* mutations
Pasupuleti et al., 2021, [133]	Obesity	Observational cohort study using the UK Biobank, including 47,466 unrelated participants who were free of T2D at baseline	✓ CHIP was present in 5.8% of participants, most frequently in *DNMT3A* (3.7%) and *TET2* (1.0%); large clones (VAF > 10%) were found in 2.4% ✓ CHIP carriers had higher waist-to-hip ratios (WHR), with prevalence increasing across WHR quintiles ✓ In obese mouse models, CHIP mutations drove rapid myeloid expansion, severe MPN/AML, and CVD ✓ CHIP-induced pathology was linked to pro-inflammatory cytokines (IL-1β, IL-6, TNF-α) and increased intracellular Ca^2+^ in HSC/Ps ✓ Drug combination (metformin, nifedipine, MCC950, anakinra) lowered organ weights, blood glucose, and reduced atherosclerotic lesions, providing cardiovascular protection	Obesity is strongly linked to CHIP in humans, and targeting CHIP-mutant cells with a combination of metformin, nifedipine, MCC950, or anakinra may offer a safe, cost-effective strategy to mitigate CHIP-related cardiovascular complications
Denicolò et al., 2022, [122]	CKD	Nested case–control study within 1419 PROVALID participants to investigate whether CHIP influences incidence or progression of DKD	✓ CHIP prevalence was estimated at 28.9% (95% CI 22.9–34.9%) ✓ Unlike established risk factors (albuminuria, HbA1c, HF, smoking) and elevated microinflammation, CHIP was not associated with incident or progressive DKD (HR 1.06; 95% CI 0.57–1.96)	In T2D, common risk factors and elevated microinflammation, but not CHIP, were linked to kidney function decline
Agrawal et al., 2022, [47]	Gout	Observational cohort study using data from 177,824 participants in the MGB Biobank and UK Biobank to investigate the association between CHIP and gout	✓ In both cohorts, gout frequency was higher among CHIP carriers ✓ In MGBB, CHIP with VAF ≥ 2% was associated with OR 1.69 (95% CI 1.09–2.61; *p* = 0.0189) ✓ In UKB, CHIP with VAF ≥ 10% had OR 1.25 (95% CI 1.05–1.50; *p* = 0.0133) ✓ Individuals with CHIP and VAF ≥ 10% had a higher risk of incident gout in UKB (HR 1.28; 95% CI 1.06–1.55; *p* = 0.0107) ✓ In *TET2* knockout mouse models, hematopoietic cells produced excess IL-1β and developed paw edema upon MSU crystal administration ✓ *TET2*-deficient macrophages secreted higher IL-1β in vitro, which was reduced by genetic or pharmacologic NLRP3 inflammasome inhibition	*TET2*-mutant CHIP is linked to a higher risk of gout and acts as an enhancer of NLRP3-mediated inflammation in response to MSU crystals
Lee et al., 2025, [129]	COPD	Prospective observational cohort study including 125 patients with COPD enrolled between 2013 and 2023	✓ CHIP prevalence was 25.6% ✓ Higher smoking intensity and COPD Assessment Test (CAT) scores were linked to an increased likelihood of CHIP positivity, particularly in current smokers ✓ CHIP in specific genes was associated with a higher risk of mild and total acute exacerbations ✓ *ASXL1* mutations were linked to a lower risk of total acute exacerbations (adjusted OR 0.19; 95% CI 0.04–0.95) ✓ CHIP status was not significantly associated with longitudinal lung function changes	In COPD patients, CHIP positivity was linked to a higher risk of acute exacerbations but did not affect long-term lung function decline
Schenz et al., 2022, [131]	COVID-19	Observational cohort study of 90 hospitalized COVID-19 patients	✓ CHIP prevalence was 37.8%, higher than expected based on median age (17%) ✓ CHIP was associated with a higher risk of hospitalization for COVID-19, but did not affect outcomes independently of age among hospitalized patients ✓ In younger patients (45–65 years), CHIP was linked to persistent lymphopenia ✓ In older patients (>65 years), CHIP-positive individuals developed long-term neutrophilia ✓ The independent effect of CHIP on cardiac biomarker elevation could not be determined	CHIP is associated with an increased risk of severe COVID-19 requiring hospitalization and with altered cellular immune responses to SARS-CoV-2, suggesting that CHIP status could serve as a biomarker for risk stratification and early treatment guidance
Bick et al., 2022, [132]	HIV	Case–control study comparing 600 PLWH from the Swiss HIV Cohort Study (SHCS) with 8111 controls from the ARIC study	✓ HIV infection was associated with a twofold higher prevalence of CHIP compared with controls (SHCS 7% vs. ARIC 3%; *p* = 0.005) ✓ ASXL1 was the most frequently mutated CHIP gene in PLWH	CHIP may play a role in the elevated cardiovascular risk observed in PLWH

Abbreviations: ASXL1: additional sex combs like 1; CHIP: clonal hematopoiesis of indeterminate potential; CKD: chronic kidney disease; COPD: chronic obstructive pulmonary disease; COVID-19: coronavirus disease 2019; DKD: diabetic kidney disease; HIV: human immunodeficiency virus; IL-1β: interleukin-1 beta; MASLD: metabolic dysfunction-associated steatotic liver disease; PLWH: people living with HIV; T2D: type 2 diabetes; TET2: ten-eleven translocation 2.

**Table 4:** Interventional trials investigating CHIP in cardiometabolic disorders.

Trial/Intervention	Target and Mechanism	Population (CHIP Enrichment)	Design/Phase	Primary Endpoints	Status	Key Notes
NCT06097663: DFV890 (oral NLRP3 inhibitor) or MAS825 (bispecific IL-1β/IL-18 mAb) vs. placebo	Inflammasome and upstream IL-1 family blockade	Coronary heart disease + CHIP (DNMT3A or TET2; VAF ≥ 2%)	Randomized, placebo-controlled; Phase 2a; ~28 participants	Change in inflammatory markers (IL-6, IL-18, hsCRP) over 12 weeks	Completed (record updated 10 December 2024)	First prospective, CHIP-enriched cardiometabolic trial; informs biomarker-driven strategies.
CANTOS: Canakinumab (50, 150, 300 mg SC q3mo) vs. placebo [158]	IL-1β neutralization	Patients with prior MI and elevated hsCRP (≥2 mg/L); CHIP not specifically enriched	Randomized, double-blind, placebo-controlled; Phase 3; 10,061 participants	MACE (nonfatal MI, nonfatal stroke, CV death)	Completed	150 mg dose significantly reduced recurrent CV events; effect independent of lipid lowering; increased risk of fatal infection; no effect on all-cause mortality.
CANTOS: Canakinumab vs. placebo [21]	IL-1β neutralization	Patients with prior MI, elevated hsCRP (>0.2 mg/dL), subset with CHIP (*TET2* or *DNMT3A*)	Randomized, placebo-controlled; Phase 3; subset analysis of ~338 CHIP-positive participants	MACE	Completed	A large-scale CHIP-enriched analysis in a cardiovascular outcomes trial; exploratory data suggest TET2 variant carriers may benefit more from IL-1β inhibition.
RESCUE/Ziltivekimab (CKD with inflammation) and HERMES (HFpEF/HFmrEF)-Ziltivekimab (anti-IL-6 ligand)	IL-6 axis inhibition	Not CHIP-enriched; high-inflammation cardiometabolic populations	Randomized, double-blind, Phase 2 biomarker study (RESCUE); HERMES outcomes in HFpEF/HFmrEF; monthly dosing	Biomarker reduction (hsCRP, thrombosis markers) → CV outcomes (HERMES)	Ongoing	Robust biomarker lowering; provides translational rationale for IL-6 blockade in CHIP-positive individuals with inflammatory cardiometabolic disease, though CHIP-specific data pending.
ARTEMIS (NCT06118281): post-MI trial of ziltivekimab	IL-6 axis inhibition	Not CHIP-enriched; acute coronary cohort	Randomized; details per registry	CV events post-MI	Recruiting (posted 25 June 2025)	Platform to explore CHIP as a prespecified subgroup if sequencing performed.
NCT05483010: statins (atorvastatin and rosuvastatin) in CCUS/MDS	Pleotropic anti-inflammatory and lipid-lowering; possible NLRP3 dampening	CCUS/lower-risk MDS (hematologic precursors to myeloid disease; many carry CHIP-like drivers)	Interventional; Phase 2	Hematologic/clinical outcomes (site-listed); CV effects exploratory Change in inflammatory biomarkers and VAF of somatic mutations	Recruiting	Not a pure CHIP-CVD trial, but relevant repurposing; examines whether statins modify clonal biology and downstream risks.
Mechanistic signal for metformin (preclinical/early-translational) [156,159]	Mitochondrial metabolism; potential clonal fitness reduction in DNMT3A mutants	-	-	-	-	Human trials in CHIP not yet registered; strong preclinical and translational rationale pointing to future interventional studies.
Signal for colchicine (cardiologic outcomes; preclinical CHIP model) [87,153]	Broad anti-inflammatory; inflammasome suppression	—(CV outcomes in CAD; CHIP not enriched)	RCTs in CAD (COLCOT/LoDoCo2 trials); preclinical TET2-CH atherosclerosis model	MACE reduction (clinical CAD trials)	Completed (CAD RCTs)	Prevented accelerated atherosclerosis in *TET2*-CH mice; CHIP-stratified human data awaited.
LoDoCo2 substudy: Low-dose colchicine (0.5 mg daily) vs. placebo [153]	Anti-inflammatory, NLRP3/IL-6 pathway modulation; potential suppression of CH clonal expansion	Patients with chronic CAD; CH assessed via targeted sequencing (*TET2*, *DNMT3A*, others); 854 participants	Randomized, placebo-controlled; exploratory substudy; median follow-up 25 months	CH growth and inflammatory biomarkers (hsCRP, IL-6)	Completed	Colchicine attenuated *TET2* clone expansion and reduced IL-6 increase; suggests potential for CH-modulated cardiovascular risk reduction.

Abbreviations: CAD: Coronary artery disease; CCUS: Clonal cytopenia of undetermined significance; CHIP: Clonal hematopoiesis of indeterminate potential; CKD: Chronic kidney disease; COLCOT: Colchicine Cardiovascular Outcomes Trial; CV: Cardiovascular; DNMT3A: DNA (cytosine-5)-methyltransferase 3A; HFmrEF: Heart failure with mildly reduced ejection fraction; HFpEF: Heart failure with preserved ejection fraction; hsCRP: High-sensitivity C-reactive protein; IL-1β: Interleukin-1 beta; IL-18: Interleukin-18; IL-6: Interleukin-6; LoDoCo2: Low-Dose Colchicine 2 trial; MACE: Major adverse cardiovascular events; mAb: Monoclonal antibody; MDS: Myelodysplastic syndromes; MI: Myocardial infarction; NLRP3: NLR family pyrin domain-containing 3; RCT(s): Randomized controlled trial(s); SC: Subcutaneous; TET2: tet methylcytosine dioxygenase 2; VAF: Variant allele frequency. →: leading to.

## Data Availability

No new data were created or analyzed in this study. Data sharing is not applicable to this article.

## Abbreviations

AA: aplastic Anemia; ACS: acute coronary syndrome; AF: atrial fibrillation; AIP: atherogenic index of plasma; AKI: acute kidney injury; AML: acute myeloid leukemia; AMH: anti-müllerian hormone; ASXL1: additional sex combs like 1; BCOR/BCORL1: BCL6 corepressor/BCL6 corepressor-like 1; BMI: body mass index; CABG: coronary artery bypass grafting; CAD: coronary artery disease; CAR-T: chimeric antigen receptor T-cell; CBC: complete blood count; CCRS: clonal cytopenia risk score; CCUS: clonal cytopenia of undetermined significance; COVID-19: coronavirus disease 2019; CKD: chronic kidney disease; CH: clonal hematopoiesis; CHIP: clonal hematopoiesis of indeterminate potential; CMML: chronic myelomonocytic leukemia; CMP: common myeloid progenitor; CMR: cardiac magnetic resonance; COPD: chronic obstructive pulmonary disease; CRP: C-reactive protein; CRS: cytokine release syndrome; ctDNA: circulating tumor DNA; CVD: cardiovascular disease; DC: dendritic cell; DCM: dilated cardiomyopathy; DKD: diabetic kidney disease; DNMT3A: DNA (cytosine-5)-methyltransferase 3A; eGFR: estimated glomerular filtration rate; EMT: epithelial–mesenchymal transition; GNMT: glycine N-methyltransferase; GWAS: genome-wide association study; HCC: hepatocellular carcinoma; HbA1c: hemoglobin A1c; HCT: hematopoietic cell transplantation; HF: heart failure; HFpEF: Heart failure with preserved ejection fraction; HFrEF: Heart failure with reduced ejection fraction; HIV: human immunodeficiency virus; HSC: hematopoietic stem cell; HSPC: hematopoietic stem and progenitor cell; HSCT: hematopoietic stem cell transplantation; HR: hazard ratio; hsCRP: high-sensitivity C-reactive protein; IBMFS: inherited bone marrow failure syndromes; IEAA: Intrinsic Epigenetic Age Acceleration; IFN-γ: interferon-gamma; IL-1β: Interleukin-1 beta; IL-6: Interleukin-6; IL-18: Interleukin-18; JAK-STAT: Janus kinase–signal transducer and activator of transcription; JAK2: Janus kinase 2; LAD: left anterior descending; LDL: low-density lipoprotein; LDLR: Low-density lipoprotein receptor; LVEF: left ventricular ejection fraction; mAb: monoclonal antibody; MDS: myelodysplastic syndrome; MACE: major adverse cardiovascular events; MASLD: metabolic dysfunction-associated steatotic liver disease; MGB: Mass General Brigham; MGUS: monoclonal gammopathy of undetermined significance; MI: Myocardial infarction; MN: Myeloid neoplasms; MPPs: Multipotent progenitors; MRI: Magnetic resonance imaging; MSU: Monosodium urate; mTOR: Mechanistic target of rapamycin; Myelodysplastic syndromes; MZF1: Myeloid zinc finger 1; NETs: Neutrophil extracellular traps; NGS: Next-generation sequencing; NK: Natural killer; NLRP3: NOD-like receptor family pyrin domain-containing 3; NYHA: New York Heart Association; OR: odds ratio; PAD: peripheral arterial disease; PARP: Poly(ADP-ribose) polymerase; PCI: Percutaneous coronary intervention; PLWH: People living with HIV; PPM1D: Protein phosphatase, Mg^2+^/Mn^2+^ dependent 1D; PRRT: Peptide receptor radionuclide therapy; q3-month: every three months; RBC: Red blood cells; RCT(s): Randomized controlled trial(s); RDW: Red cell distribution width; SC: Subcutaneous; SF3B1: Splicing factor 3B subunit 1; SRSF2: Serine/arginine-rich splicing factor 2; STEMI: ST-elevation myocardial infarction; STING: Stimulator of interferon genes; SULT4A1: Sulfotransferase family 4A member 1; T2D: Type 2 diabetes; TET2: Ten-eleven translocation 2; TIMP1: Tissue inhibitor of metalloproteinases-1; TNF-α: Tumor necrosis factor alpha; TNFR-2: Tumor necrosis factor receptor 2; TOPMed: Trans-Omics for Precision Medicine; TP53: Tumor protein p53; U2AF1: U2 small nuclear RNA auxiliary factor 1; VAF: Variant allele frequency; WHI: Women’s Health Initiative; WBC: white blood cells; WHR: Waist-to-hip ratio.
